# Nanoparticle Delivery of Alu RNA Adjuvants Enhances Vaccine Immunogenicity

**DOI:** 10.1021/acsami.5c16047

**Published:** 2025-08-28

**Authors:** Alexander J. Kwiatkowski, Jacob A. Schulman, Hayden M. Pagendarm, Lucinda E. Pastora, John T. Tossberg, Ruilin Zhang, Neil C. Chada, Mia E. Woodruff, Taylor L. Sheehy, Karan Arora, John Karijolich, Thomas M. Aune, John T. Wilson

**Affiliations:** † Department of Chemical and Biomolecular Engineering, 5718 Vanderbilt University, Nashville, Tennessee 37235, United States; ‡ Department of Biomedical Engineering, Vanderbilt University, Nashville, Tennessee 37235, United States; § Department of Medicine, Vanderbilt University Medical Center, Nashville, Tennessee 37232, United States; ∥ Department of Pathology, Microbiology, and Immunology, 12328 Vanderbilt University Medical Center, Nashville, Tennessee 37232, United States; ⊥ Vanderbilt Institute for Infection, Immunology, and Inflammation, Vanderbilt University Medical Center, Nashville, Tennessee 37232, United States; # Vanderbilt Institute of Chemical Biology, Vanderbilt University Medical Center, Nashville, Tennessee 37232, United States; ∇ Vanderbilt Center for Immunobiology, Vanderbilt University Medical Center, Nashville, Tennessee 37232, United States; ○ Vanderbilt-Ingram Cancer Center, Vanderbilt University Medical Center, Nashville, Tennessee 37232, United States

**Keywords:** adjuvant, A-to-I editing, Alu RNA, nanoparticles, endosomal escape

## Abstract

Vaccine adjuvants stimulate innate immunity to enhance and shape adaptive immune responses. However, approved adjuvants typically elicit weak CD8^+^ T cell responses to protein- and peptide-based vaccines, motivating an investigation into the discovery and testing of new adjuvants. Unedited forms of endogenous Alu RNAs are sensed by pattern recognition receptors (PRRs) to trigger sterile inflammation, and therefore we hypothesized that synthetic Alu RNA molecules could be harnessed as vaccine adjuvants. To enhance their intracellular delivery, Alu RNA was copackaged with a model antigen into polymer nanoparticles that promoted endosomal escape of Alu RNA to the cytosol. Using this nanovaccine formulation, we found that Alu RNA activated antigen-presenting cells in vitro and in vaccine-site draining lymph nodes in vivo. Furthermore, we demonstrated that vaccine formulations containing Alu RNA as an adjuvant elicited comparable CD8^+^ T cell responses to those containing the common but highly heterogeneous RNA adjuvant PolyIC, and that this response protected mice from tumor challenge. Based on this, we further evaluated the antitumor efficacy of nanovaccine formulations containing Alu RNA adjuvants in mice with established tumors, again observing comparable responses to formulations containing PolyIC. Finally, we found that nanovaccines adjuvanted with Alu RNA could improve responses to anti-PD-1 immune checkpoint blockade in tumor-bearing mice. Overall, this study demonstrates that unedited Alu RNA coformulated with antigen in polymer nanoparticles can be harnessed as an effective vaccine adjuvant for stimulating CD8^+^ T cell responses with antitumor function and may offer a sequence-defined alternative to PolyIC.

## Graphical abstract

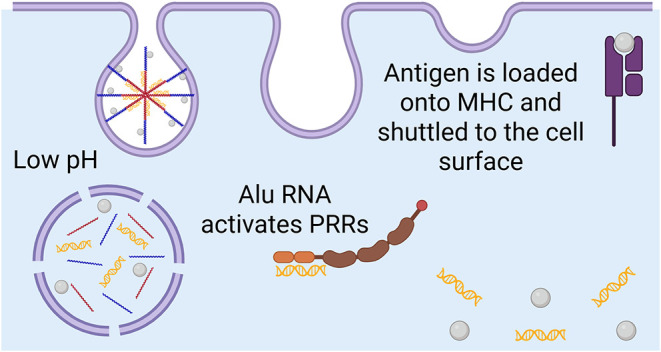

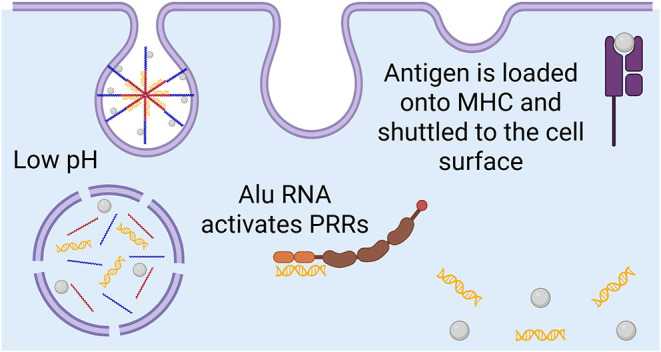

## 1 Introduction

Stimulating CD8^+^ cytotoxic T cell responses is important to the efficacy of vaccines against a number of infectious pathogens as well as for therapeutic cancer vaccines targeting tumor antigens.^1,2^ Activating a CD8^+^ T cell response requires antigen presentation on major histocompatibility complex (MHC)-I in the context of appropriate immunostimulatory cues that enhance the expression of costimulatory molecules (e.g., CD86) by dendritic cells (DCs) and the secretion of cytokines that support T cell differentiation and effector function.^3–7^ Achieving this requires the addition of vaccine adjuvants that activate innate immune responses, which augment and shape the subsequent adaptive immune response, including T cell responses. Specifically, professional antigen-presenting cells (APCs) present antigen in a pro-inflammatory context to naïve CD8^+^ T cells that differentiate to acquire their cytotoxic effector function. However, most approved vaccine adjuvants (e.g., alum, MF59) are largely ineffective at stimulating CD8^+^ T cell responses, particularly against protein and peptide antigens that have inherently low immunogenicity.^8,9^ This challenge has fueled decades of research focused on the development and testing of new adjuvants for stimulating cellular immunity.^10–12^ While several classes of immunostimulatory molecules have been explored as adjuvants, nucleic acids have demonstrated significant promise due to their ability to activate pattern recognition receptors (PRRs) that trigger the expression of pro-inflammatory cytokines that support cytotoxic T lymphocyte (CTL) activation and differentiation.^13–15^ PRRs can sense foreign nucleic acids using toll-like receptors (TLRs; e.g., TLR-3, 7, 9), melanoma differentiation-associated protein-5 (MDA-5), retinoic acid-inducible gene-I (RIG-I), and cyclic GMP-AMP synthase (cGAS),^16,17^ which trigger overlapping but distinctive transcriptional programs primarily via nuclear factor kappa B (NF-κB) and interferon-regulatory factor (IRF) signaling that dictate the phenotype of the innate immune response. Notable examples of advanced nucleic acid adjuvants include CpG DNA (CpG 1018), a TLR-9 agonist that is a component of the Heplisav-B vaccine, and polyinosinic/polycytidylic acid (PolyIC), a double-stranded RNA agonist of TLR3 and MDA-5 that has been widely explored in recent clinical trials of cancer vaccines.^18–20^

At homeostasis, PRRs must distinguish between foreign and self-RNA and do so primarily based on unique chemical or structural features characteristic of RNA molecules. For example, RNA can undergo 5′ capping or adenosine-to-inosine (A-to-I) editing to avoid detection by RNA sensors such as RIG-I and MDA-5.^21^ A-to-I editing is a post-translational modification where RNA-specific adenosine deaminase 1 (ADAR1) converts adenosine to inosine, covalently marking endogenous dsRNA and blocking its detection by cytosolic PRRs such as MDA-5.^22–24^ Defects in ADAR1 can lead to impaired A-to-I editing that results in stimulation of PRRs and eliciting autoinflammatory responses that contribute to chronic inflammatory and autoimmune diseases (e.g., multiple sclerosis).^25^ By contrast, elevated levels of A-to-I editing in cancer cells have been associated with reduced tumor immunogenicity and a decreased immune infiltrate into the tumor.^26–28^ Accordingly, inhibition of RNA editing has recently been explored for cancer immunotherapy.^29,30^ For example, ADAR1 knockdown resulted in increased type-I interferon (IFN-I) production in triple-negative breast cancer^31^ and combining CRISPR knockout of ADAR1 with immune checkpoint blockade (ICB) resulted in improved response compared to ICB alone in treating B16 melanoma,^32^ potentially positioning ADAR1 inhibitors or modulation of A-to-I editing as therapeutic options to overcome resistance to ICB. Additionally, treatment with a small-molecule ADAR1 inhibitor suppressed tumor growth in a preclinical model of prostate cancer.^33^

One class of RNA that typically undergoes A-to-I editing is Alu RNA. Alu elements are short interspersed nuclear elements found in introns and can be divided into two subclasses, the less immunogenic AluJ class, and the more immunogenic AluS class, with both classes being largely immunologically inert in their edited form.^34,35^ However, in diseases such as multiple sclerosis, Alzheimer’s disease, and inflammatory bowel disease, there is a concomitant increase in unedited double-stranded Alu RNA that contributes to elevated IFN-I production.^25,36,37^ There are multiple Alu RNAs within each class, and we have previously established that the unedited forms of Alu elements can stimulate immune cell activation through RIG-I and TLR3 resulting in downstream upregulation of genes including *Relb*, *Il6*, *Ddx58*, and *Ifit5*.^36,37^ Notably, in vitro studies found that AluJb (chr17:76,418,582–76,418,856 on the GrCh37/hg19 assembly) can stimulate IFN-I and NF-κB responses, while Alu5 (chr1:36,944,168–36,944,489 on the GrCh37/hg19 assembly) predominantly results in IFN-I production.^38^ Consistent with their immunostimulatory properties, we have previously shown that intratumoral administration of unedited synthetic Alu RNAs delayed tumor growth and prolonged survival in a CD8^+^ T cell-dependent manner.^39^ Here, we seek to parlay this activity into the use of Alu RNAs as vaccine adjuvants for stimulating the cellular immunity to vaccines.

We therefore hypothesized that AluJb and Alu5 RNA could be harnessed as vaccine adjuvants to improve cellular immune responses to protein antigens, which are poorly immunogenic when they are delivered alone or with conventional adjuvants. However, Alu RNA predominantly activates cytosolic RNA sensors such as RIG-I and MDA-5, yet the intracellular delivery of free, unmodified RNA is highly inefficient due to rapid clearance, poor cellular uptake, and degradation in the endosome.^40,41^ Additionally, we and others have demonstrated that simple coadministration of mixtures of antigen and nucleic acid adjuvants often only minimally improves immune responses since free antigen and adjuvant do not efficiently reach the same APCs or the correct subcellular compartment.^42–48^ To address these barriers, we have previously described a nanoparticle (NP) vaccine delivery system that allows for electrostatic loading of nucleic acid adjuvants and covalent conjugation of protein antigens (Figure 1), a dual-delivery strategy that increases the probability that both vaccine components are internalized by the same APCs, thereby dramatically improving immune responses.^49^ The NP is assembled using diblock copolymers (Figure 1A) where the first block consists of 90% of the hydrophilic monomer (poly­(ethylene glycol)) methacrylate (PEGMA) copolymerized with 10% pyridyl disulfide ethyl methacrylate (PDSMA). PEGMA provides a hydrophilic corona to promote NP stability and integrity, and the PDSMA groups enable the conjugation of thiolated antigen to the NP surface via the thiol-disulfide exchange reaction (Figure 1B). The second block of the polymer consists of a 50:50 molar ratio of dimethylamino ethyl methacrylate (DMAEMA) and butyl methacrylate (BMA). This design allows for electrostatic complexation of nucleic acids to cationic DMAEMA groups and promotes endosomal escape of antigen and adjuvant cargo to the cytosol, which we have demonstrated increases antigen presentation on MHC-I and enables access to cytosolic nucleic acid sensing pathways (e.g., RIG-I, cGAS) (Figure 1C).^49^ We previously used this platform for improving the adjuvant activity of PolyIC, a poorly defined and heterogeneous nucleic acid adjuvant that suffers from potential toxicities and high batch-to-batch variability.^50,51^ By contrast, Alu5 and AluJb RNA are well-defined synthetic analogs of endogenous Alu elements, but, to our knowledge, have not been explored as vaccine adjuvants. Herein, we leverage our nanovaccine platform to evaluate Alu5 and AluJb RNA as nucleic acid adjuvants and demonstrate their capacity to enhance CD8^+^ T cell responses to protein antigens to a similar degree as PolyIC. Additionally, we demonstrate the potential application of this nanovaccine formulation—Alu Vax—in the context of cancer vaccines, a promising and emerging modality in immuno-oncology.^16,52,53^

**Figure 1:**
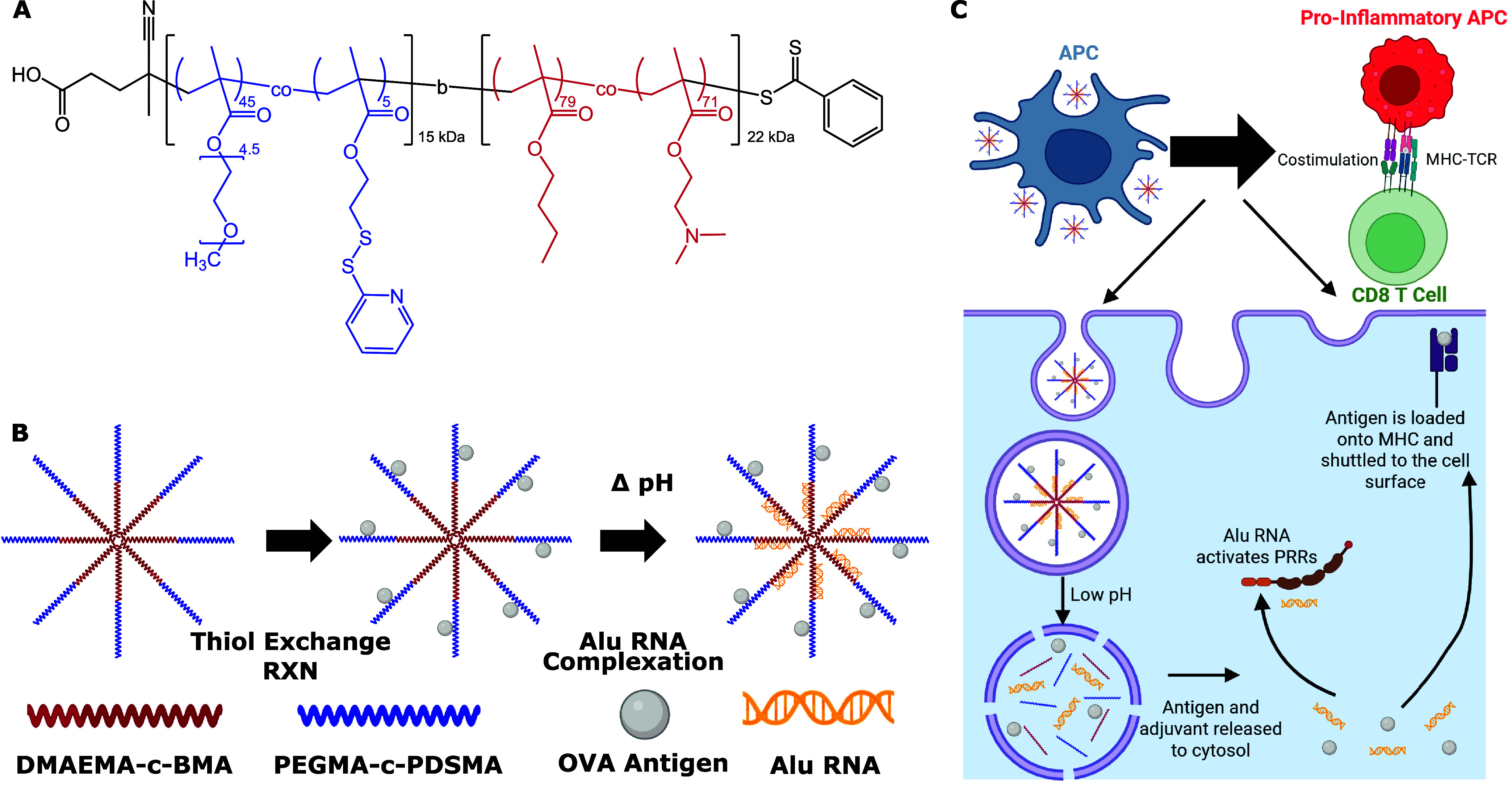
Nanoparticles for dual delivery of antigen and Alu RNA adjuvants. A) The chemical structure of the (PEGMA_90_-*c*-PDSMA_10_)-*b*-(DMAEMA_50_-*c*-BMA_50_) polymer used for coloading of antigen and nucleic acid adjuvants into NPs that enhance intracellular delivery. B) The process of loading NPs with antigen and nucleic acid adjuvant. Thiolated OVA antigen was covalently integrated into the NP via disulfide exchange with PDS groups. Alu RNA was then complexed to the DMAEMA-*c*-BMA block at low pH followed by neutralization of the pH to form the nanovaccine. C) Proposed mechanism of intracellular antigen and adjuvant delivery. Antigen-presenting cells endocytose NPs, which are trafficked to acidic endolysosomes. The decreased pH protonates DMAEMA groups and triggers endosomal escape of antigen and RNA cargo into the cytosol. Alu RNA then activates PRRs, while OVA antigen is processed for loading on MHC-I molecules for presentation to CD8^+^ T cells.

## 2 Materials and Methods

### 2.1 Polymer Synthesis and Characterization

Amphiphilic diblock copolymer poly­[(polyethylene glycol) methacrylate)_0.9_-*co*-(pyridyl disulfide ethyl methacrylate)_0.1_]_13.7 kDa_-*block*-[(dimethylamino ethyl methacrylate)_0.5_-*co*-(butyl methacrylate)_0.5_]_25 kDa_ was synthesized via reversable addition–fragmentation chain transfer (RAFT) polymerization, using 2,2′-azobis­(4-methoxy-2,4-dimethylvaleronitrile) (V-70) (Wako Chemicals, Richmond, VA) as the initiator and 4-cyano-4-(phenyl-carbonothioylthio) pentanoic acid as the chain transfer agent (CTA). Aluminum oxide (activated, basic, Brockmann I; Sigma-Aldrich) gravity filtration columns removed inhibitors from monomer stocks prior to polymerization.

PEGMA (Mw = 300 Da, Sigma-Aldrich) and PDSMA were synthesized as previously described.^49^ PEGMA and PDSMA were reacted under argon for 10 min in dioxane (40 wt % monomer and CTA) and then reacted overnight at 40 °C in an oil bath. To form a macroCTA, PEGMA and PDSMA were reacted at a 90:10 molar ratio with an initial monomer:CTA:initiator ratio of 55:1:0.2. The macroCTA was then purified via dialysis in a 3.5 kDa MWCO SnakeSkin membrane against pure acetone 3×, 1× 50:50 acetone:DI water, and 1× pure DI water, with each step lasting ∼8 h. The product was then frozen overnight and lyophilized for a minimum of 72 h.

MacroCTA was then polymerized with DMAEMA (Sigma-Aldrich) and BMA (Sigma-Aldrich) to form the resultant polymer (Figure 1A). DMAEMA (50%) and BMA (50%) ([M]­0/[mCTA]­0 = 143) were added to the macroCTA dissolved in dioxane (30 wt % monomer and macroCTA) along with V-70 initiator ([mCTA]­0/[I]­0 = 5) and allowed to react under argon for 15 min followed by incubation overnight in a 40 °C oil bath. The polymer was then dialyzed and lyophilized as described for the macroCTA. ^1^H nuclear magnetic resonance (NMR) (CDCl3 with TMS; Bruker AV400 spectrometer) and gel permeation chromatography (GPC) were used to determine the degree of polymerization, theoretical molecular weight, and polymer composition (Figures S1 and S2).

### 2.2 Nanoparticle Formulation and Characterization

The polymer was dissolved in ethanol at 50 mg/mL and diluted to 10 mg/mL in phosphate buffer pH = 7.4. When diluted, the solution was pipetted to form micellar NPs and incubated for 20 min at room temperature. Prior to injection into mice, the NPs were buffer exchanged and concentrated using four cycles of centrifugal dialysis. Briefly, the NPs were centrifuged at 14,000 *g* for 15 min in Amicon, 3 kDa MWCO filters (Millipore) with 475 μL of PBS added to the filter between cycles. The NP concentration was measured using the absorbance at 310 nm via a spectrophotometer (Synergy H1Multi-Mode Microplate Reader, BioTek).

Endotoxin-free EndoFit Ovalbumin (OVA) (Invivogen) was used as the model antigen. The OVA antigen was conjugated to the NPs using a thiol exchange reaction to conjugate the thiolated free amines on the OVA to the pendant PDS groups of the NPs (Figure 1B). Thiolation was induced by incubating the NPs and OVA for 1 h in the presence of ∼24 molar excess of 2-iminothiolane (Traut’s Reagent, Thermo Fisher Scientific) in reaction buffer (100 mM phosphate buffer, pH 8, supplemented with 1 mM EDTA), as previously described.^49^ Centrifugation through Zeba desalting columns (0.5 mL, 7 kDa MWCO, Thermo Fisher Scientific) was used to remove excess Traut’s Reagent. Thiolation efficiency was measured using Ellman’s reagent (Thermo Fisher Scientific) to ensure a molar ratio of ∼2–6 thiols/OVA was incorporated.

Thiolated OVA was then reacted overnight with NPs at an 8:1 ratio (NP:OVA) in PBS to form OVA-NPs. SDS-polyacrylamide gel electrophoresis (SDS-PAGE) was used to verify antigen conjugation using 4–20% Mini-Protean TGX Precast protein gels (Bio-Rad). Gels were run at 130 V for 1 h and then read on the Gel Doc EZ System (Bio-Rad). To evaluate conjugation efficiency, we utilized fluorescently labeled (AlexaFluor647 (AF647); excitation: 650 nm, emission: 665 nm; Thermo Fisher Scientific) at a 2:1 AF647:OVA molar ratio. OVA was allowed to incubate with NHS-Ester AF647 overnight, and then free dye was removed via 7 cycles of centrifugal dialysis at 14,000 *g* for 15 min in 30 kDa MWCO Amicon filters (Millipore).

OVA-NPs or NPs were then complexed with the adjuvant at a 6:1 charge ratio in citrate buffer pH 4 for 45 min at room temperature. After incubation, the pH was raised using 3× the volume of phosphate buffer. Agarose gel retardation was then used to verify the complexation by comparing free RNA to RNA-NP complexes. SYBR Safe was added to the 2% agarose solution at a 1:10,000 dilution prior to gel solidification. 0.5 μg of RNA was loaded into each well and the gel was run for 1 h at 100 V then immediately imaged on a Gel Doc EZ system (Bio-Rad). Loading efficiency was quantified via the Quant-it RiboGreen Reagent and RNA Assay Kit (Thermo Fisher) which was conducted according to the manufacturer’s suggested protocol. The hydrodynamic diameter of all NPs was measured on a Malvern Zetasizer Ultra (Malvern).

### 2.3 Cell Culture

THP1-Dual monocytes were cultured in Roswell Park Memorial Institute medium (RPMI 1640) (Gibco) supplemented with 25 mmol/L HEPES, 2 mmol/L l-glutamine, 10% heat-inactivated Fetal Bovine Serum (HI FBS) (Gibco), 100 U/mL penicillin, 100 μg/mL streptomycin (Gibco), and 100 μg/mL Normocin. A549-Dual lung carcinoma cells were cultured in Dulbecco’s Modified Eagle Medium (DMEM) (Gibco) supplemented with 2 mM l-glutamine, 4.5 g/L d-glucose, 10% HI FBS (Gibco), and 100 U/mL penicillin/100 μg/mL streptomycin (Gibco). For both reporter cell lines, the selection agents blasticidin and zeocin were added every other passage at 10 and 100 μg/mL, respectively. SIINFEKL-expressing (OVA_257–264_) MC38-OVA cells were cultured in DMEM supplemented with 4.5 g/L glucose, 2 mmol/L l-glutamine, 10% HI FBS (Gibco), 100 U/mL penicillin, and 100 μg/mL streptomycin. MC38-OVA cells were tested for mycoplasma prior to injection in mice.

Bone marrow was harvested from the femurs and tibias of 8–10-week-old mice. Following euthanasia, bones were excised from the mice and transferred into a biological safety cabinet, where bone marrow was flushed out of the bones using 5 mL of media per bone through a 25G needle. Bone marrow was mechanically pushed through a 70 μm strainer and centrifuged at 380 *g* for 5 min. The supernatant was aspirated, and cells were resuspended in 3 mL of ACK lysis buffer (KD Medical) for 3 min at room temperature, then centrifuged at 380 *g* for 5 min. Cells were rinsed in 5 mL of media and counted, then plated in a 100 mm^2^ Petri dish. For macrophage differentiation, 5 × 10^6^ cells were plated, and for DC differentiation, 2 × 10^6^ cells were plated, with both cell types being plated in a total of 15 mL of media. Cells were cultured in RPMI 1640 with 2 mM l-glutamine, 10 mM HEPES, 100 U/mL penicillin, 100 μg/mL streptomycin, 1 mM sodium pyruvate, 1% nonessential amino acids, 10% heat-inactivated FBS, and 56 mM β-mercaptoethanol. Macrophages received 20 ng/mL macrophage colony-stimulating factor (M-CSF), while DCs received 20 ng/mL granulocyte-macrophage colony-stimulating factor (GM-CSF). On day 3, 5 mL of media was added with cytokine. On days 5 and 7, half of the media was collected and centrifuged at 380 *g* for 5 min. A new dish was prepared if cells were >80% confluent. If not, the cells were placed back in the initial dish. On day 10, cells were dissociated from the plate and plated in a 12 well plate in 1 mL of media. Cells were treated with NPs and collected for flow cytometry 24 h post-treatment.

### 2.4 Reporter Cell Assays

A549-Dual cells were dissociated from the flask using 0.05% trypsin, centrifuged at 380 *g* for 5 min, and then resuspended in a known volume of media for counting. After the cells were counted, they were diluted to a concentration of 500,000 cells/mL, and 50,000 cells per well were plated in a 96-well plate. THP1-Dual cells were in suspension, so these cells were collected and counted and 50,000 cells per well were plated in 100 μL of media. The next day, cells were treated with NPs, and assays were conducted 24 h post-treatment. These cells produce luciferase downstream of IRF and secreted alkaline phosphatase (SEAP) downstream of NF-κB, and Quanti-Luc and Quanti-Blue (Invivogen) assays were performed according to the manufacturer’s protocol to evaluate these metrics. Briefly, 30 μL of supernatant was transferred to a white plate, a spectrophotometer (Synergy H1Multi-Mode Microplate Reader, BioTek) dispensed 50 μL of the Quanti-Luc luciferase reagent per well, and the luminescence was immediately read. Additionally, 20 μL of supernatant was added to 180 μL of Quanti-Blue SEAP detection reagent and incubated at 37 °C for 1 h, and then absorbance was read at 620 nm on a spectrophotometer (Synergy H1Multi-Mode Microplate Reader, BioTek). For both assays, data were normalized to the highest value, and a dose-response curve was fit using a four-parameter model in GraphPad Prism.

### 2.5 Mice and Injections

All experiments conducted in mice were approved by the Vanderbilt University Institutional Animal Care and Use Committee (IACUC) and conducted according to the guidelines set forth by the IACUC. Studies utilized 8–10-week-old female mice; except for studies evaluating NP trafficking, mice were injected subcutaneously with NPs at the base of the tail along the midline with a dose of 58 μg of OVA and 30 μg of adjuvant. For trafficking studies, injections were made to the right of the midline at the base of the tail with a dose-matched treatment using AF647-OVA.

### 2.6 In Vivo Imaging

To determine trafficking kinetics away from the injection site, an IVIS Lumina III (PerkinElmer) was used to evaluate the presence of AF647-OVA ex vivo. Briefly, mice were injected subcutaneously to the right of the midline at the base of the tail with NPs containing 58 μg of AF647-OVA and 30 μg of AluJb or a matched dose of free AF647-OVA. Mice were euthanized at 6 or 24 h post-treatment injection, and the draining inguinal lymph node and contralateral axillary lymph node were harvested. Images were taken using excitation and emission wavelengths of 650 and 665 nm, respectively. Analysis was conducted using Living Image software (PerkinElmer) by drawing ROIs around the lymph nodes to quantify fluorescence (total flux).

### 2.7 Tissue Harvest and Processing

Spleens were excised, adipose tissue was removed, and then mechanically dissociated through a 70 μm strainer using a syringe plunger. Cells were centrifuged at 380 *g* for 5 min, and the supernatant was discarded before resuspending in 3 mL of ACK lysis buffer (KD Medical) for 5 min at room temperature. Following centrifugation at 380 *g* for 5 min, cells were resuspended in 5 mL of RPMI 1640 media and centrifuged in the same settings. After the supernatant was removed, cells were resuspended in RPMI 1640 media, and ∼4–6 million cells were transferred to a 96-well plate for flow cytometry. Lymph nodes were collected 24 h post-treatment and dissociated as previously described for spleens, and all cells were then plated for flow cytometry. Blood was collected via cardiac puncture through a 25G needle. The needle was removed, and the blood was placed in a tube containing EDTA buffer that was centrifuged for 15 min at 2000 *g*. Serum was collected and placed at −20 °C until titer evaluation.

### 2.8 Flow Cytometry

For the analysis of the dLN and bone marrow-derived cells, cells were rinsed in flow buffer (PBS with 2% FBS and 0.05% sodium azide) and centrifuged at 380 *g* for 5 min. Cells were resuspended in 50 μL of media containing 1 μg of Fc Block for 15 min at 4 °C, and then 50 μL of media containing the surface stain antibodies was added for 30 min at 4 °C. Cells were placed in 200 μL of FoxP3 transcription factor fixation solution (ThermoFisher) for 1 h at room temperature. This was followed by centrifugation at 650 *g* for 5 min in 200 μL of permeabilization buffer (eBioscience). Intracellular antibodies were added in 100 μL of permeabilization buffer for 30 min at room temperature. Cells were centrifuged at 650 *g* for 5 min, resuspended in 200 μL of flow buffer, centrifuged again, and resuspended in 150 μL of flow buffer before running on a Cytek Aurora (Cytek Biosciences).

To evaluate cell response in the spleen, cells were resuspended in 100 μL of media containing 5 μL of AF647 labeled tetramer for SIINFEKL–H2-K^b^. After centrifugation at 380 *g* for 5 min, cells were Fc-blocked and surface-stained as described for lymph nodes. Cells were then centrifuged for 5 min at 380 *g*, subsequently fixed in 200 μL of 2% paraformaldehyde for 10 min at room temperature, and then centrifuged at 650 *g* for 5 min. Cells were resuspended in 200 μL of flow buffer and centrifuged again before resuspending in 150 μL of flow buffer before running on a Cytek Aurora (Cytek Biosciences).

### 2.9 IFN ELISA

IFN-α and IFN-β ELISA kits were purchased from Invivogen and conducted according to the manufacturer’s recommended protocol. Briefly, plates were coated with 50 μL of 5 μg/mL capture antibody and allowed to incubate overnight. The capture antibody was removed, and plates were blocked with 3% BSA + 0.05% Tween 20 for 2 h at 37 °C. After the blocking buffer was removed, 50 μL of BMDC or BMDM supernatant was added to the well followed by the immediate addition of 50 μL of 30 ng/mL Lucia-conjugated detection antibody. Following a 2 h incubation at 37 °C plates were washed 3× with a plate washer. 50 μL of Quanti-Luc 4 was added to the plate, and luminescence was immediately evaluated.

### 2.10 Quantitative PCR

Mice were injected with vaccines containing 100 μg of adjuvant, and lymph nodes were collected 24 h later and placed in RNALater (Invitrogen) overnight. The tissue was transferred to RLT Plus lysis buffer and dissociated using a TissueLyser II instrument (Qiagen). RNA was then extracted using an RNeasy Plus Mini Kit (Qiagen) on a QIAcube (Qiagen). RNA was reverse transcribed to cDNA using an iScript cDNA synthesis kit (Bio-Rad) according to the manufacturer’s suggested protocol. RT-PCR was conducted on a Bio-Rad CFX Connect Real-Time System using TaqMan primers and a master mix. Primers used included Cxcl10 (Mm00445235), IL6 (Mm00446190), IL12 (Mm00434174), and TNF (Mm00443258) with Hmbs (Mm001143545) used as a housekeeping gene. Fold change was calculated as $2^{-({Cq}_{\mathrm{Hmbs}}‐Cq_{geneofinterest})}$ .

### 2.11 Antibody Titer

Nunc MaxiSorp plates (high-protein-binding plates; Thermo Fisher Scientific) were coated with 100 μL of 5 μg/mL of OVA overnight at room temperature. The coating antibody was removed, and plates were blocked in 0.01% BSA in PBS + 0.05% Tween 20 for 20 min, then rinsed 5× on an ELx50 plate washer (BioTek) with PBS + 0.05% Tween 20. This process was then repeated using a SuperBlock (Thermo Fisher ). 12.5 μL of serum diluted 1:10 in 0.01% BSA in PBS + 0.05% Tween 20 was added to the first column of the plate and mixed with 100 μL of PBS + 0.05% Tween 20. This initial dilution was subsequently diluted 1:5 10 times, and samples were incubated for 2 h at room temperature and then washed 5× using a plate washer. Subsequently, 100 μL of 0.005 μg/mL IgG1 or 0.1 μg/mL IgG2c was added to the plate, incubated for 1 h at room temperature, and then rinsed 5× using a plate washer. 100 μL of streptavidin-horseradish peroxidase at a 1:20,000 dilution was added for 30 min at room temperature and then rinsed on a plate washer 5×. 100 μL of SureBlue Reserve TMB 1 peroxidase substrate (KPL) was added, and 5 min later the reaction was quenched with 100 μL of 1 M HCl and absorbance was immediately measured at 450 nm. Titers were determined using reciprocal dilutions and a sigmoidal fit in GraphPad Prism by determining when the A_450 nm_ value was equal to the mean + two standard deviations of the serum of PBS-treated mice.

### 2.12 Tumor Studies

Two days before tumor inoculation, the right hind flank was shaved and hair removal cream was applied to allow for tumor visualization. On the day of tumor inoculation, once ∼40–80% confluency was reached, cells were resuspended in sterile PBS at 1 × 10^7^ cells/mL. Mice were anesthetized with isoflurane and 1 × 10^6^ cells were injected subcutaneously in the right flank through a 20G needle. Importantly, to reduce the risk of shearing, cells were drawn up into the syringe without the needle attached to avoid passing through the needle twice.

For prophylactic studies, NPs containing 58 μg of OVA and 30 μg of adjuvant were injected subcutaneously along the midline of the back at the base of the tail with booster injections on 7 and 14 days post-treatment initiation. Seventeen days after the completion of the treatment regimen, mice were inoculated with tumors. Alternatively for therapeutic vaccination studies, mice were inoculated with tumors and once the tumors reached a volume of ∼65 mm^3^, treatment was administered subcutaneously with booster injections on day 7 and 14 post-treatment. For studies involving combination therapy with ICB, 100 μg of anti-PD-1 was administered intraperitoneally in 100 μL of sterile PBS, starting on day 0 and every third day until day 15 post-treatment initiation at tumor volume of ∼80 mm^3^.

### 2.13 Antibodies

TCR-β (H57-597, eFluor 450), CD4 (RM4-5, BV605, BioLegend), CD44 (IM7, PerCP, BioLegend), CD366/Tim3 (RMT3-23, PE-Dazzle594, BioLegend), CD223/LAG3 (C9B7W, BV785, BioLegend), CD279/PD-1 (29F.1A12, BV510, BioLegend), CD8α (53–6.7, AF488, BioLegend), CD69 (H1.2F3, PE-Cy7, BioLegend), CD62L (MEL-14, BV711, BioLegend) *K*_i_-67 (SolA15, AF532, eBioscience), Granzyme B (NGZB, PE-Cy5.5, eBioscience), KLRG1 (2F1, SB645, eBioscience), TCR-β (S33-966, eF450, eBioscience), and FoxP3 (FJK-16S, PE, eBioscience). Fixable Viability Dye (eF780, eBioscience), CD11b (BV510, M1/70, BioLegend), CD11c (BV711, N418, BioLegend), F4/80 (AF488, BM8, BioLegend), CD206 (BV605, C068C2, BioLegend), Arg1 (PE, A1exF5, eBioscience), MHC-II (BV421, M5/114.15.2, BioLegend), CD80 (PerCP-eF710, 16-10A1, eBioscience), CD86 (PerCP, GL-1, BioLegend), GR-1 (PE-Cy7, RB6-8C5, BioLegend), and Nos2 (AF532, CXNFT, eBioscience).

### 2.14 Statistical Analyses

GraphPad Prism (version 10.4) was used to conduct all statistical analyses. Significance levels were defined as follows: * represents *p* < 0.05, ** represents *p* < 0.01, *** represents *p* < 0.001, and **** represents *p* < 0.0001 and are denoted in the figure legend. Comparisons between more than two groups utilized a one-way ANOVA, while analyses conducted over time (i.e., tumor growth) used a two-way ANOVA. Tukey’s post hoc test was used to compare between groups. Plotted data are represented as mean ± one standard error of the mean. Survival statistics were determined using a Kaplan–Meier curve and the log-rank (Mantel-Cox) test.

## 3 Results

### 3.1 Formulation of Nanovaccines for Dual Delivery of Antigen and Alu RNA Adjuvants

We first sought to explore whether our polymeric nanocarrier platform was amenable to the loading of Alu RNA and could enhance its immunostimulatory potency for potential use as a vaccine adjuvant. We synthesized (PEGMA-*c*-PDSMA)-*b*-(DMAEMA-*c*-BMA) diblock copolymers via RAFT polymerization as previously described.^49^ Diblock copolymers were first dissolved in ethanol at 50 mg/mL and then diluted to 10 mg/mL in PBS to form micelles and mixed with thiolated antigen, here OVA, for covalent antigen loading. The pH of the solution was then dropped to 4.2 and Alu5, AluJb, or PolyIC was added to promote electrostatic complexation, followed by neutralization of the solution by adding 3× the volume of phosphate buffer 100 mM pH = 7 (Figure 1B). Nanovaccine formulations containing both the OVA and Alu RNA will be termed “Alu Vax,” while formulations without the OVA antigen but loaded with Alu will be termed NP/Alu and the OVA conjugated to NPs without adjuvants will be referred to as OVA-NPs. As expected, DLS revealed an increase in NP size with the loading of nucleic acid adjuvants (Figure 2A), with OVA-NPs ∼10 nm in diameter and loaded NPs were ∼30 nm in diameter (Figure 2A). SDS-PAGE confirmed near-complete conjugation of AF647-labeled OVA as indicated by impaired OVA migration and a dramatic reduction in the free OVA band (Figure 2B). Densitometry revealed over 80% conjugation for all groups with no adjuvant-dependent impact on conjugation. Furthermore, agarose gel electrophoresis confirmed near-complete complexation of both Alu5 and AluJb to the NPs (Figure 2C), which, when quantified via Ribogreen assay, was over 90% for all adjuvants. Hence, NPs enable facile and efficient copackaging of the OVA and Alu RNA, yielding a nanovaccine formulation for evaluating Alu elements as adjuvants.

**Figure 2:**
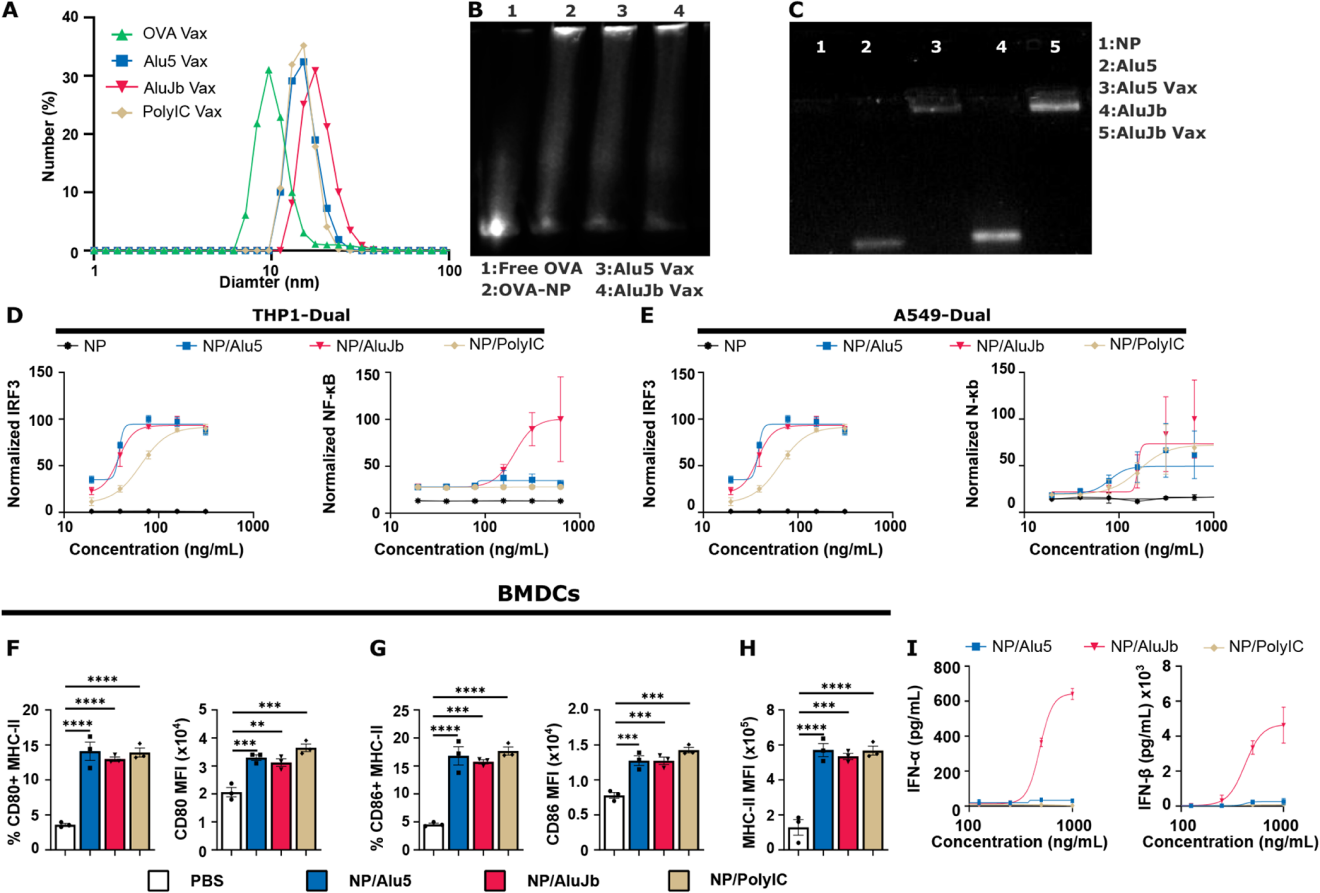
Characterization of nanoparticle vaccines with Alu RNA adjuvants. A) NP size distribution measured by DLS. B) SDS-PAGE gel validating AF647-OVA conjugation. Lanes are as follows: 1: Free OVA, 2: OVA-NP, 3: Alu5 Vax, and 4: AluJb Vax. C) Agarose gel validating Alu RNA complexation. Lanes are as follows: 1) NP alone, 2) Free Alu5, 3) Alu5 Vax, 4) Free AluJb, 5) AluJb Vax. Dose–response curves for IRF and NF-κB activation in D) THP1-Dual monocyte and E) A549-Dual lung carcinoma reporter cells. Expression levels of F) CD80, G) CD86, and H) MHC-II on BMDCs following treatment with the indicated NP/RNA complex. I) Type-I interferon concentration in culture supernatant following treatment of BMDCs with NP/RNA complexes (*n* = 3 per group, and experiments were performed twice). *p < 0.05, **p < 0.01, ***p < 0.001, and ****p < 0.0001. *P* values were determined by one-way ANOVA with Tukey’s post hoc test to compare the mean of the indicated groups.

### 3.2 Alu RNA-Loaded NPs Activate Reporter Cells and Bone Marrow-Derived APCs

To evaluate the immunostimulatory effects of Alu elements delivered using polymeric NPs, we initially assessed their ability to induce IRF and NF-κB responses in THP1-Dual monocyte and A549-Dual lung carcinoma reporter cells and compared them to the widely used nucleic acid adjuvant PolyIC. Both cell lines have a secreted luciferase reporter of the IRF pathway and a SEAP reporter of NF-κB signaling. We observed that NP/AluJb produced the highest levels of IRF activation (Figure 2D); however, EC_50_ values for NP/AluJb, NP/Alu5, and NP/PolyIC were relatively similar. Similar trends were observed with respect to the NF-κB response with NP/AluJb inducing a response, while NP/Alu5 and NP/PolyIC produced minimal NF-κB responses (Figure 2D). In A549-Dual reporter cells, NP/Alu5 and NP/AluJb stimulated comparable IRF responses that were greater than those induced by NP/PolyIC (Figure 2E). Interestingly, NP/Alu5 and NP/PolyIC stimulated comparable levels of NF-κB, but both were lower than NP/AluJb suggesting that AluJb, can activate multiple pathways while Alu5 is limited to inducing IRF responses. Notably, unloaded NPs did not elicit IRF or NF-κB responses in either cell line, suggesting that the effects are dependent on adjuvant cargo (Figure 2D,E). Taken together, these data suggest that NP/Alu5 has similar immunostimulatory abilites to NP/PolyIC in vitro, with NP/AluJb tending to exert stronger effects than NP/Alu5 and with NP/AluJb having a slight bias toward NF-κB signaling.

DCs are important to stimulating immune responses to vaccines, and so we next sought to determine the ability of Alu RNA to activate murine bone marrow-derived DCs (BMDCs) by measuring the expression of the activation markers MHC-II, CD80, and CD86. NP/Alu5, NP/AluJb, and NP/PolyIC increased the frequencies of CD80^+^MHC-II^+^ and CD86^+^MHC-II^+^ cells to a comparable degree (Figures 2F–H and S3C,D). Interestingly, NP/AluJb elicited significantly stronger IFN-α and IFN-β responses compared to NP/Alu5 and NP/PolyIC (Figure 2I). Similar effects were observed when treating bone marrow-derived macrophages (BMDMs) (Figures S3A–D and S4E,F). Taken together, these data show that the delivery of Alu5 and AluJb with endosomolytic NPs activates BMDCs and BMDMs, supporting their potential use as vaccine adjuvants.

### 3.3 AluJb NPs Traffic to the Draining Lymph Node and Induce APC Activation

Effective vaccination requires the transport of antigenic cargo to the dLN for the priming of CD8^+^ T cells. To evaluate antigen distribution to the dLN, we injected mice subcutaneously with AluJb Vax formulated with AF647-OVA or dose-matched free AF647-OVA for ex vivo analysis of lymph node (LN) fluorescence 6 and 24 h postadministration (Figure 3A). Administration of AF647-AluJb Vax resulted in increased fluorescent signal in the draining LN (dLN) (inguinal LN) at 24 h post-treatment compared to free AF647-OVA, which did not accumulate to a significant degree (Figure 3B). No increase in fluorescent signal was observed in the nondraining axillary lymph node at either time point (Figure 3B). The increased accumulation at 24 h but not 6 h suggests that AluJb Vax drains to the lymph node with slower kinetics than what would be expected with passive transport via the lymphatics.^42,54^

**Figure 3:**
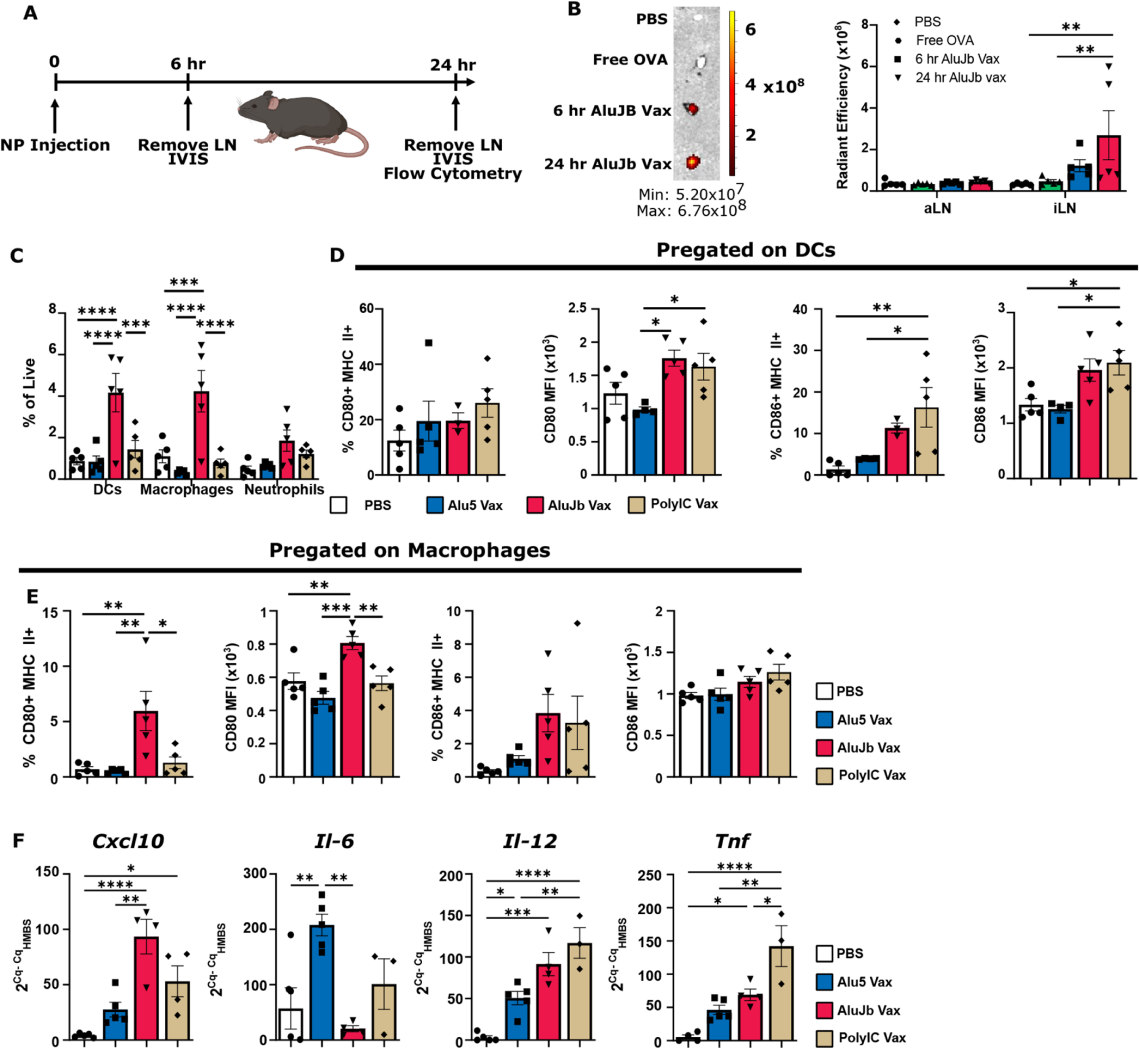
Alu RNA-loaded nanoparticle vaccines traffic to the dLN to activate APCs. A) Timeline of experiments evaluating AluJb Vax distribution and cellular uptake in the vaccine site dLN. B) Representative fluorescent IVIS images of the inguinal draining lymph node (iLN) and quantification of the total flux in the axillary (aLN) and iLN (*n* = 4–5 per group). *P* values were determined by two-way ANOVA with Tukey’s post hoc test. **p < 0.01. C) Quantification of the frequency of DCs, macrophages, and neutrophils in the iLN. *P* values were determined by two-way ANOVA with Tukey’s post hoc test. ***p < 0.001 and **** *p* < 0.0001. D) Assessment of CD80, CD86, and MHC-II expression levels on DCs in the dLN. E) Evaluation of the expression of CD80, CD86, and MHC-II by macrophages in the dLN (*n* = 3–5 per group). *P* values were determined by one-way ANOVA with Tukey’s post hoc test. *p < 0.05, ** *p* < 0.01, and ***p < 0.001. F) Determination of cytokine levels via RT-PCR (*n* = 3–5 per group). *P* values were determined by one-way ANOVA with Tukey’s post hoc test. *p < 0.05, ** *p* < 0.01, ***p < 0.001, and **** *p* < 0.0001.

Based on this improved accumulation in the lymph node, we also evaluated changes in the myeloid cell composition of the dLN using flow cytometry. Notably, AluJb Vax increased the frequency of CD11c^+^ DCs with a concomitant increase in CD11b^+^F4/80^+^ macrophage frequency compared to Alu5 Vax, OVA-PolyIC-NP, or PBS-treated mice (Figures 3C and S5A–C). Innate immune cells need to be activated to elicit a CD8^+^ T cell response. To this end, we evaluated the activation state of CD11c^+^ DCs in the dLN, finding that AluJb Vax and PolyIC Vax increased median fluorescent intensity (MFI) of CD80 compared to Alu5 Vax, while only PolyIC Vax significantly increased the frequency of CD86^+^ MHC-II^+^ DCs and levels of CD86 as assessed by MFI (Figures 3D, S5, S6A, and S7). Interestingly, the inverse effect was seen in macrophages, where AluJb Vax more effectively increased the frequency of CD80^+^ MHC-II^+^ cells compared to all other formulations (Figure 3E). However, the frequency of CD86^+^ MHC-II^+^ macrophages did not significantly change with any of the treatments (Figures 3E and S6B). Furthermore, each vaccine formulation elicited elevated levels of distinct cytokines, with AluJb Vax, Alu5 Vax, and PolyIC Vax increasing levels of *Cxcl10*, *Il-6*, and *Tnf*, respectively (Figure 3F). Interestingly, all formulations increased *Il-12* production (Figure 3F), where *Il-12* is implicated in T cell activation. Overall, these data suggest that Alu RNA-loaded NPs accumulate in the vaccine site-draining (inguinal) lymph node, where AluJb Vax increased the frequency of DCs and macrophages in the draining lymph node and activated DCs in a manner analogous to that of PolyIC Vax, while AluJb Vax more effectively activated macrophages with a cytokine profile varying with adjuvant.

### 3.4 Alu Vax Increases Antigen-Specific CD8^+^ T Cells and T Cell Activation in the Spleen

Having established the impact of Alu Vax on APCs, we next sought to evaluate the adjuvant effects on T cell responses elicited by vaccination. Mice were immunized on days 0, 7, and 14, and we assessed the frequency of SIINFEKL/H2–K^b^ tetramer-positive T cells in the spleen on day 21 (Figure 4A). Immunization with Alu5 Vax, AluJb Vax, and PolyIC Vax increased the frequency of tetramer-positive CD8^+^ T cells to a similar extent (Figures 4B, S8A, and S9) with an increased frequency of antigen-specific (i.e., tetramer-positive) CD62L^–^CD44^+^ effector memory T cells (T_EM_) relative to CD62L^+^CD44^+^ central memory T cells (T_CM_), which were also the same for all formulations (Figure 4C). While we did not assess antigen-specific CD4^+^ T cell responses, we characterized changes in the overall frequency of CD4^+^ T_EM_ cells and T_CM_, observing that AluJb Vax and PolyIC Vax significantly shifted the CD4^+^ compartment toward a T_EM_ phenotype compared to PBS-treated mice (Figures 4D and S8C), whereas Alu5 Vax did not modulate CD4^+^ T cells to a state different from PBS-treated mice (Figure 4D). Finally, we also measured anti-OVA IgG1 and IgG2c antibody titers to assess the ability of Alu adjuvants to modulate the B cell response and as an indication of Th1/2 bias as IgG1 and IgG2c titers are linked with T helper cell type 2 (Th_2_) and Th_1_ responses, respectively. Treatment with Alu5 Vax resulted in a stronger IgG1 response than PolyIC Vax (Figure 4E), while all adjuvants produced similar, but lower, IgG2c titers (Figure 4E). Taken together, these data indicate that Alu5 and AluJb RNA have comparable adjuvant effects on T cells to the widely used PolyIC when delivered with OVA using polymeric NPs, with AluJb RNA potentially acting as a slightly more Th1-biased adjuvant relative to Alu5.

**Figure 4:**
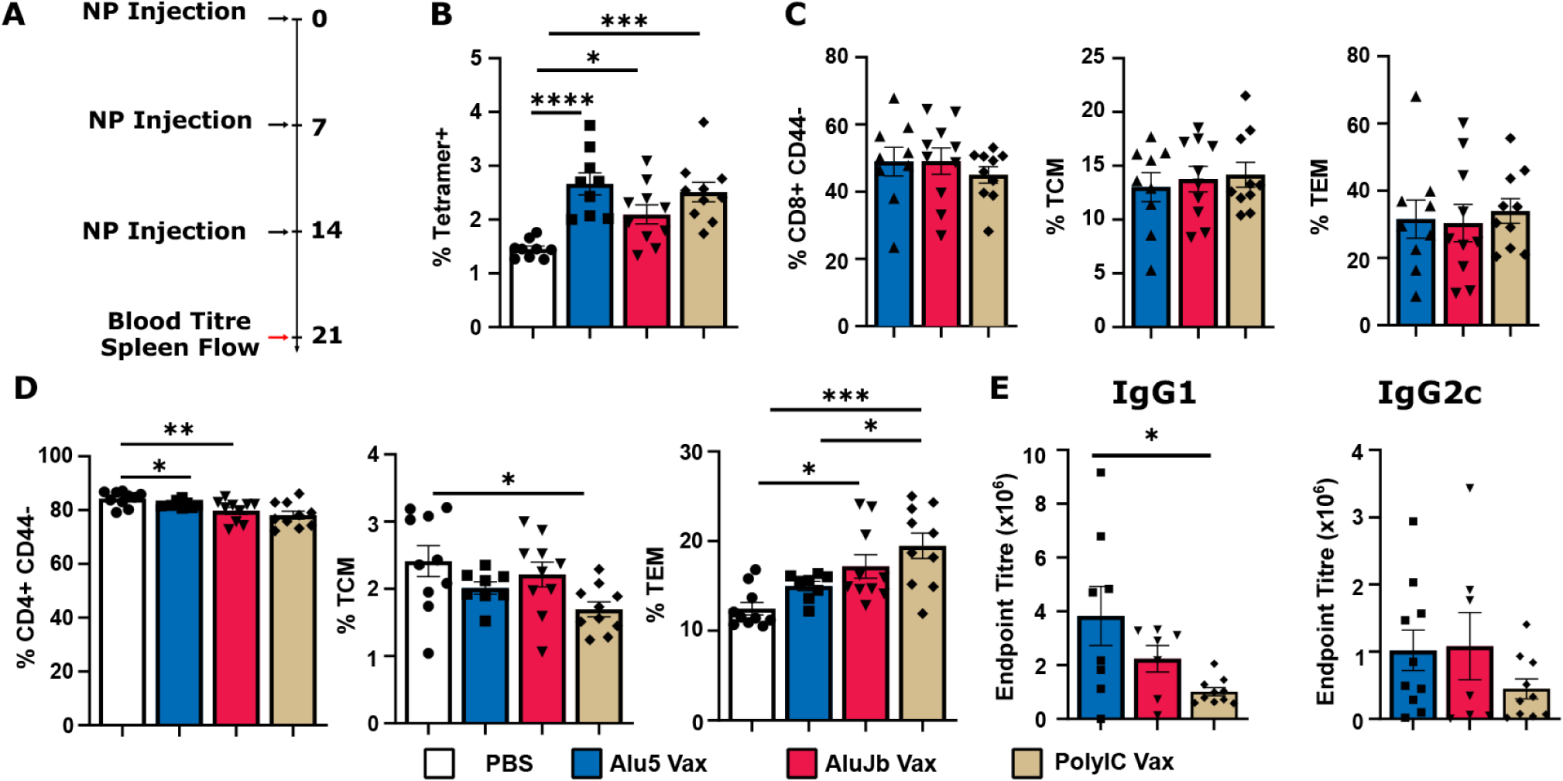
Alu adjuvants increase immune responses to nanoparticle vaccine. A) Timeline of immunizations and experiments at the study endpoint. B) Percent of CD8^+^ T cells positive for SIINFEKL/H-2Kb tetramer^+^ cells in the spleen following three NP vaccinations. C) Evaluation of the memory and effector states of CD8^+^ T cells in the spleen. D) Assessment of effector and memory states of CD4^+^ T cells in the spleen. E) Determination of IgG titer in the blood following three NP vaccinations. *n* = 7–10 per group. *P* values were determined by one-way ANOVA with Tukey’s post hoc test. *p < 0.05, **p < 0.01, *** *p* < 0.001, and ****p < 0.0001.

### 3.5 Alu RNAs Are Effective as Cancer Vaccine Adjuvants

Based on the adjuvant effects of Alu RNAs in enhancing cellular immunity, we next assessed their capacity to protect against tumor development as a metric of T cell effector function. Mice were administered weekly prophylactic vaccinations of Alu5 Vax, AluJb Vax, and PolyIC Vax for 3 weeks, then challenged subcutaneously with SIINFEKL-expressing (OVA_257–264_) MC38 (MC38-OVA) colon cancer cells 17 days later (Figure 5A); OVA-NP conjugates lacking a nucleic acid adjuvant were administered as a control. Vaccination with Alu5 Vax, AluJb Vax, PolyIC Vax, and OVA-NP resulted in smaller tumors on days 15–23 post-tumor inoculation compared to PBS treatment, and AluJb Vax and PolyIC Vax had significantly smaller tumors than that of OVA-NP on day 21 post-inoculation (Figure 5B). Alu5 Vax, AluJb Vax, and PolyIC Vax significantly extended survival compared to mice treated with unadjuvanted OVA-NPs or PBS, with each adjuvanted formulation conferring 80% complete protection from tumor challenge (Figure 5C). Importantly, the administration of NP vaccination did not result in significant weight loss, indicating that vaccine formulations do not result in systemic toxicity (Figure 5D). To better assess the potential of Alu RNA adjuvants for cancer vaccines, we assessed efficacy in a more clinically relevant therapeutic setting where mice with ∼70 mm^3^ MC38-OVA tumors received weekly vaccinations for 3 weeks (Figure 5E). Additional control groups in this study included OVA-NPs complexed to the A-to-I edited versions of Alu5 and AluJb, which have base pair changes from A-to-I to decrease their ability to activate PRRs.^38^ Edited forms of Alu5 and AluJb have been previously demonstrated to lack immunostimulatory effects,^25,36,38,39^ and, therefore, were postulated to be ineffective as adjuvants. NPs delivering edited RNAs had limited effect on tumor progression, whereas Alu5 Vax significantly extended median survival to 16 days post-treatment initiation compared to 10 days with Edited Alu5 Vax (Figure 5F,G). Treatment with Alu5 Vax and AluJb Vax extended median survival to 16 and 22 days compared to 9 days in PBS-treated mice without causing weight loss (Figure 5H). Finally, we combined our vaccine formulations with the clinically used ICB anti-PD-1, initiating treatment at a tumor volume of 80 mm^3^ (Figure 5I). Alu5 Vax and AluJb Vax combined with ICB provided a survival benefit over PBS and ICB alone (Figure 5J,K). Although not statistically significant, AluJb Vax + ICB nearly doubled median survival compared to Alu5 Vax + ICB, with median survival times of 28 and 16 days, respectively (Figure 5J,K). Overall, these studies suggest that all adjuvants perform analogously in a prophylactic setting, but AluJb may be a superior adjuvant for cancer vaccines based on improved response when combined with ICB.

**Figure 5:**
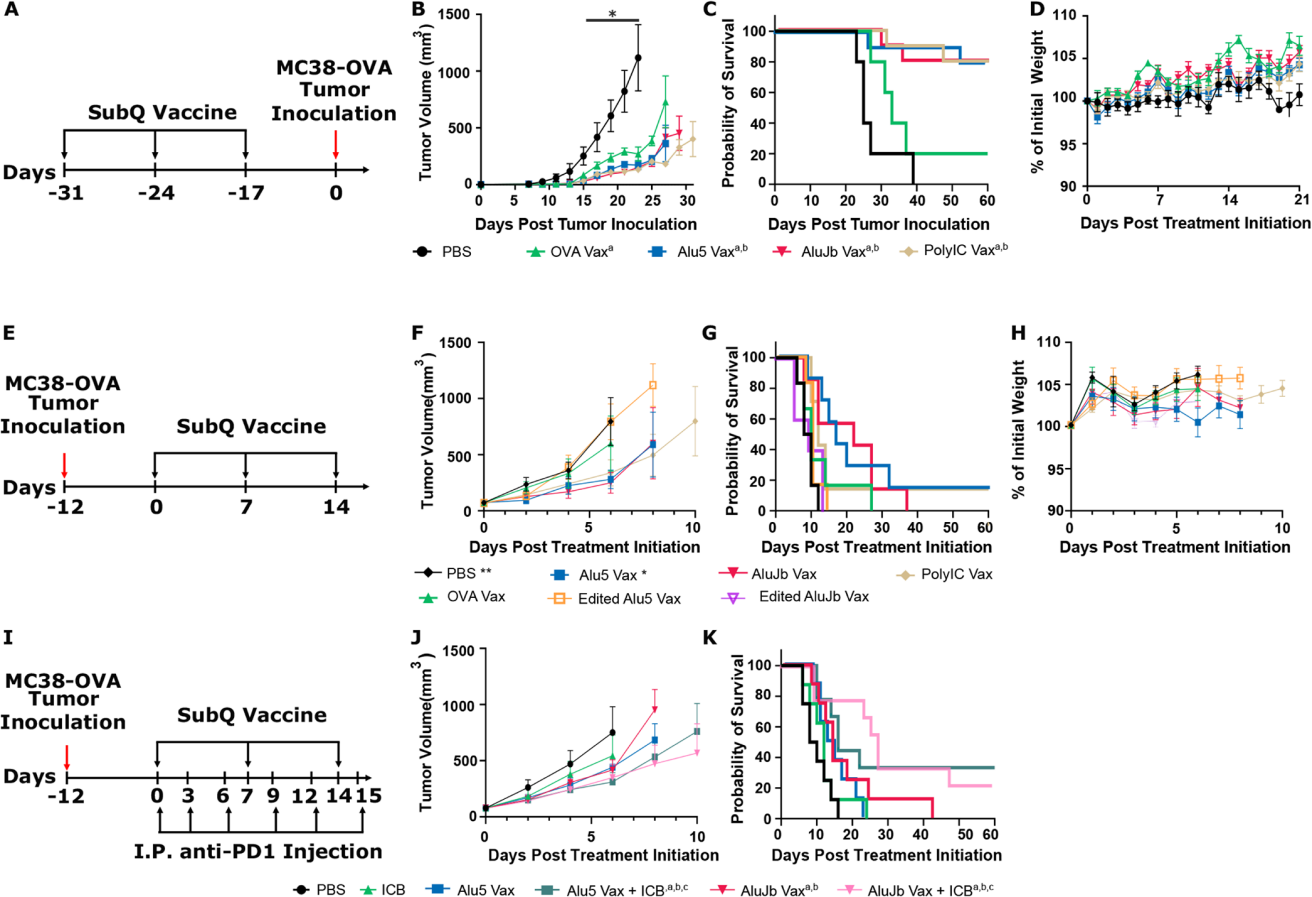
Alu RNA shows efficacy as cancer vaccine adjuvants. A) Prophylactic vaccination schedule and tumor challenge timeline. B) Tumor growth curves and C) Kaplan–Meier survival plots for mice challenged with MC38-OVA cells following vaccination with the indicated formulation. ^a^ denotes statistically significant survival differences from PBS, and ^b^ represents statistically significant differences from OVA-NP. D) Tracking of weight loss following the initiation of vaccination. E) Timeline of tumor inoculation and therapeutic vaccination. F) Tumor growth curves and G) Kaplan–Meier survival plots for mice with MC38-OVA cells treated with the indicated vaccine. H) Evaluation of weight loss following therapeutic vaccination. I) Timeline of the study combining vaccination and anti-PD-1 immune checkpoint blockade. J) Tumor growth curves survival and K) Kaplan–Meier survival plots for mice with MC38-OVA cells treated with the indicated vaccine and anti-PD-1 ICB (*n*= 7–10). *p < 0.05, **p < 0.01, and ***p < 0.001, ^a^ represents significant differences from PBS, ^b^ represents significant differences from ICB, and ^c^represents significantly different from NPs without ICB. *P* values were determined by one-way ANOVA with Tukey’s post hoc test and survival statistics were determined using a Kaplan–Meier curve and the log-rank (Mantel-Cox) test.

## 4 Discussion

Defects in A-to-I editing of Alu RNA can lead to self-RNA sensing via PRRs that stimulate innate immune responses that enhance and shape cellular immunity. For example, sensing of unedited Alu RNA has been implicated in the progression of autoimmune disease, whereas some cancers exhibit elevated levels of A-to-I editing to suppress inflammatory responses and avoid immune detection.^55,56^ Here, we postulated that such immunostimulatory Alu RNAs could be effectively “repurposed” as vaccine adjuvants. To test this, we leveraged a nanovaccine platform developed by our group that enabled dual delivery of a subunit antigen (OVA) and Alu RNA.^49^ Using this system, we found that both AluJb and Alu5 RNA exhibited adjuvant properties comparable to those of the well-established adjuvant PolyIC, resulting in the generation of CD8^+^ T cell responses that enhanced responses to immune checkpoint blockade in a murine cancer model. To our knowledge, this represents the first description of the use of Alu RNA sequences as vaccine adjuvants and establishes proof-of-concept to motivate future immunological characterization and further optimization of their immunostimulatory properties.

Consistent with our previous work,^25,36,38^ we found that AluJb was more potent than Alu5 with respect to inducing both NF-κB and IRF signaling in immune cells, which largely correlated with adjuvant effects on DCs and macrophages in the vaccine site-draining lymph node. However, prime-boost-boost immunization with Alu5 Vax, AluJb Vax, and PolyIC Vax resulted in similar CD8^+^ T cell responses, which may reflect the high immunogenicity of OVA and/or different cytokine responses induced by each adjuvant, leading to similar adaptive immune responses. The modest differences in antigen-specific CD8^+^ T cell responses could be a result of modification of the lysine in SIINFEKL during the thiolation process, such that the antigen delivered is slightly different from what is present on the cancer cells. Nonetheless, differences in IgG1 and IgG2c titers were observed between the adjuvants, with AluJb RNA eliciting a more Th1-biased response compared to Alu5 RNA, which may reflect a differential capacity of each Alu RNA to direct CD4^+^ T cell responses and to modulate the Th1/Th2 balance, with implications for vaccine design. Additional investigation is needed to more fully determine the differential effects of AluJb and Alu5 adjuvants on CD4^+^ T cells and humoral responses.

While the protective and therapeutic efficacy of both Alu RNA adjuvants was comparable in tumor models, there was a slight benefit of using AluJb over Alu5 RNA, which resulted in a modest 6-day survival benefit when used as a therapeutic cancer vaccine but conferred a near doubling in median survival from 16 to 28 days when combined with ICB. Therefore, based on its immunostimulatory properties and modest benefits in inhibiting tumor growth, AluJb RNA may hold promise as an adjuvant for therapeutic cancer vaccines. Notably, AluJb RNA can be enzymatically produced and is a chemically defined RNA molecule (i.e., defined sequence and length), and, therefore, may serve as an alternative to PolyIC, which has been used extensively in clinical trials as an adjuvant for cancer vaccines but suffers from heterogeneity and toxicity.^19,50,51^ Interestingly, Alu RNAs are only found in primates,^57,58^ which may result in less potent induction of immune responses and therefore the need for elevated doses in higher species. Therefore, future work to advance Alu RNAs as cancer vaccine adjuvants should include the characterization of antigen-specific and bystander T cell responses in the tumor microenvironment (TME) following therapeutic vaccination. Additionally, their capacity to enhance T cell responses to tumor antigens (e.g., neoantigenic peptides) rather than the model antigen OVA should be evaluated not only to increase translational relevance but also to discern whether Alu RNA adjuvants elicit different responses when combined with less immunogenic antigens. Finally, beyond cancer applications, there are also opportunities to assess Alu RNA as adjuvants for infectious diseases, where the selection of a specific type of Alu RNA may enable control over IgG class switching, as necessary for specific pathogens. In summary, our studies show that AluJb and Alu5 RNA can be used as effective vaccine adjuvants with the potential to tailor immune responses through control of the RNA sequence and structure.

## 5 Conclusions

We have shown that unedited Alu RNA elements can be harnessed as effective vaccine adjuvants when codelivered with a subunit antigen using an endosome-destabilizing polymer NP. Vaccines formulated with Alu RNA adjuvants induce cellular immune responses capable of protecting against tumor challenge and delaying tumor growth when administered to mice with preexisting tumors. We found that the use of Alu RNA, and in particular AluJb RNA, as adjuvants, was comparable to that of the established adjuvant PolyIC in eliciting CD8^+^ T cell responses with antitumor function, positioning Alu RNA as a sequence-defined alternative to PolyIC.

## Supplementary Material



## Notes

Figure S1. Polymer NMR Figure S2. Polymer GPC Figure S3. BMDM Activation State Figure S4. BMDC and BMDM Flow Cytometry Gating Scheme Figure S5. Lymph Node Population Flow Cytometry Figure S6. Lymph Node Activation Flow Cytometry Plots Figure S7. Lymph Node Flow Cytometry Gating Scheme Figure S8. Spleen Flow Cytometry Flow Cytometry Plots Figure S9. Spleen Flow Cytometry Gating (PDF)

## Abbreviations

V-70: 2,2′-Azobis­(4-methoxy-2,4 dimethylvaleronitrile)
AF: AlexaFluor
APC: Antigen presenting cell
BMDC: Bone marrow-derived dendritic cell
BMDM: Bone marrow-derived macrophage
BMA: butyl methacrylate
CTA: Chain transfer agent
CR: Complete responder
DC: Dendritic cell
DMAEMA: dimethylamino ethyl methacrylate
dLN: Draining lymph node
DMEM: Dulbecco’s Modified Eagle Medium
GM-CSF: Granulocyte macrophage-colony stimulating factor
HI FBS: Heat-inactivated fetal bovine serum
IACUC: Institutional Animal Care and Use Committee
ICB: Immune checkpoint blockade
IFN: Interferon
m-CSF: Macrophage-colony stimulating factor
MDA5: Melanoma differentiation-associated protein 5
MHC: Major histocompatibility complex
NP: Nanoparticle
NF-κB: Nuclear factor kappa-B
NMR: Nuclear magnetic resonance
OVA: Ovalbumin
PRR: Pattern recognition receptors
PEGMA: poly­(ethylene glycol) methacrylate
PEI: polyethylenimine
PLL: poly l-lysine
PolyIC: polyinosinic:polycytidylic acid
PolyICLC: Polyinosinic-polycytidylic acid-l-lysine-carboxymethylcellulose
PDSMA: pyridyl disulfide ethyl methacrylate
RAFT: reversible addition–fragmentation chain transfer
RT-PCR: real-time-polymerase chain reaction
RPMI 1640: Roswell Park Memorial Institute medium
SDS-PAGE: Sodium dodecyl sulfate polyacrylamide gel electrophoresis
SEM: Standard error of the mean
T_CM_: T central memory cell
T_EM_: T effector memory cells
Th_1_: T helper cell type 1
Th_2_: T helper cell type 2
TLR: Toll-like receptor
TME: Tumor microenvironment
