# Supplementary material for: Nanoparticle Delivery of Alu RNA Adjuvants Enhances Vaccine Immunogenicity
Source: ACS Appl Mater Interfaces. 2025 Aug 28;17(36):50560–72. doi: 10.1021/acsami.5c16047 (PMC12442014; doi:10.1021/acsami.5c16047)
Supplement: Supplementary file 1 [file am5c16047_si_001.pdf]

Supporting Information

Nanoparticle Delivery of Alu RNA Adjuvants Enhances Vaccine Immunogenicity

Alexander J. Kwiatkowski[1], Jacob A. Schulman[2], Hayden M. Pagendarm[2], Lucinda E. Pastora[1], John T. Tossberg[3], Ruilin Zhang[4,5], Neil Chada[2], Mia E. Woodruff[2], Taylor L. Sheehy[2], Karan Arora[1], John Karjolich[4,5], Thomas M. Aune[3,4], and John T. Wilson,[1,2,5-8]*

[1]Department of Chemical and Biomolecular Engineering, Vanderbilt University Nashville, Tennessee 37235
[2]Department of Biomedical Engineering, Vanderbilt University, Nashville, Tennessee 37235
[3]Department of Medicine, Vanderbilt University Medical Center, Nashville, Tennessee 37232
[4]Department of Pathology, Microbiology, and Immunology, Vanderbilt University Medical Center, Nashville, Tennessee 37232
[5]Vanderbilt Institute for Infection, Immunology, and Inflammation, Vanderbilt University Medical Center, Nashville, Tennessee 37232
[6]Vanderbilt Institute of Chemical Biology, Vanderbilt University Medical Center, Nashville, Tennessee 37232
[7]Vanderbilt Center for Immunobiology, Vanderbilt University Medical Center, Nashville, Tennessee 37232
[8]Vanderbilt-Ingram Cancer Center, Vanderbilt University Medical Center, Nashville, Tennessee 37232

Corresponding Author Email: John.t.wilson@vanderbilt.edu

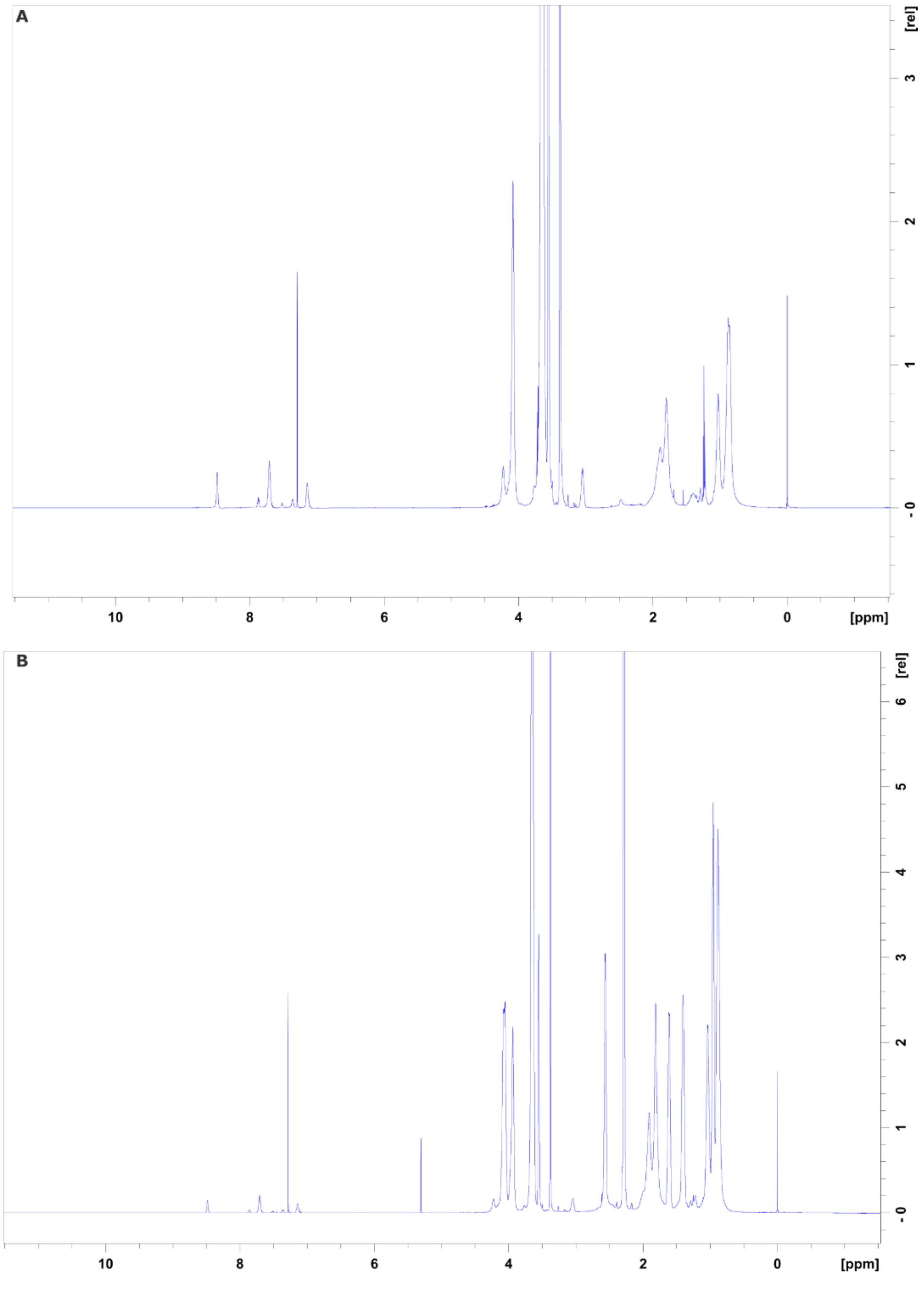

Figure S1. NMR characterization of polymers. ^{3}H NMR spectra of A) PEGMA$_{90}$-*c*-PDSMA$_{10}$ and B) (PEGMA$_{90}$-*c*-PDSMA$_{10}$)-*b*-(DMAEMA$_{50}$-c-BMA$_{50}$) copolymers.

A

B

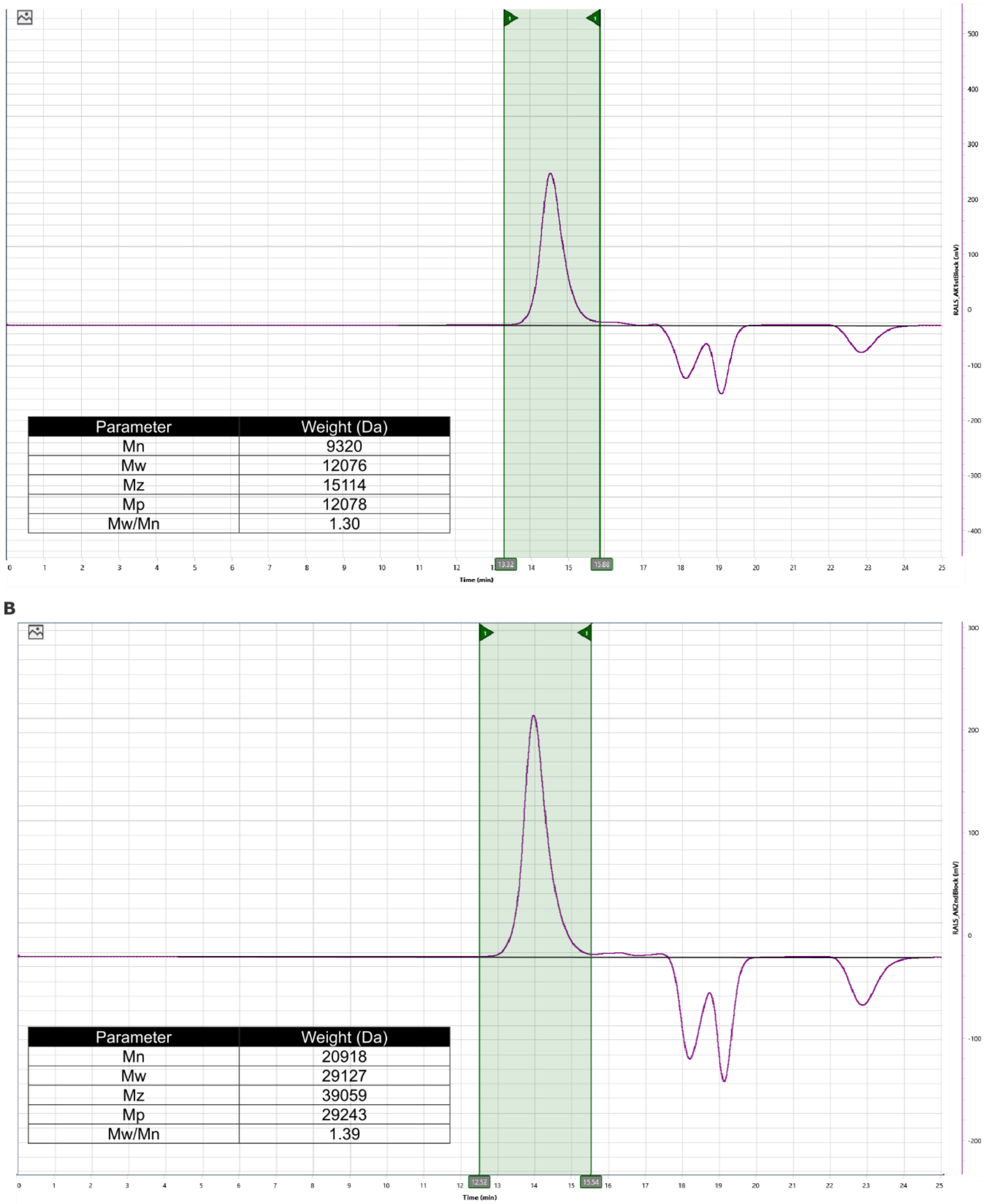

Parameter	Weight (Da)
Mn	9320
Mw	12076
Mz	15114
Mp	12078
Mw/Mn	1.30

Parameter	Weight (Da)
Mn	20918
Mw	29127
Mz	39059
Mp	29243
Mw/Mn	1.39

Figure S2. Characterization of polymers by gel permeation chromatograph (GPC). GPC chromatograms of A) $PEGMA_{90}$-*c*-$PDSMA_{10}$ and B) ($PEGMA_{90}$-*c*-$PDSMA_{10}$)-*b*-($DMAEMA_{50}$-c-BMA_{50}) copolymers.

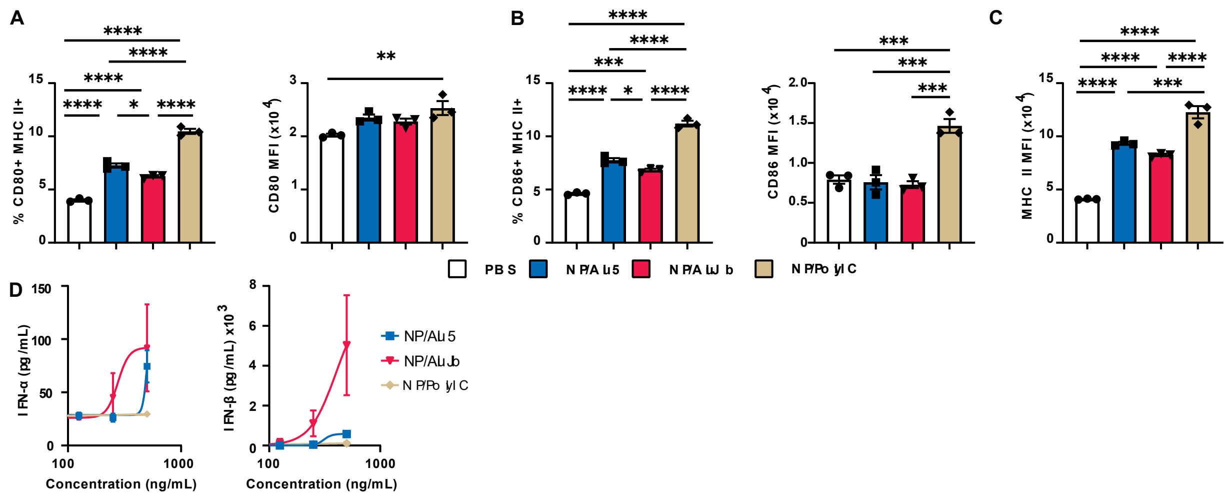

Figure S3. Alu RNAs can activate BMDMs. Expression levels of A) CD80, B) CD86, and C) MHC II on BMDMs following treatment with indicated NP/RNA complex. D) Interferon production by BMDMs after treatment with NP/RNA complexes. (*n* = 3 per group, and experiments were performed twice). *p<0.05, **p<0.01, ***p<0.001, and ****0.0001. P values determined by one-way ANOVA with Tukey's post-hoc test to compare the mean of indicated groups.

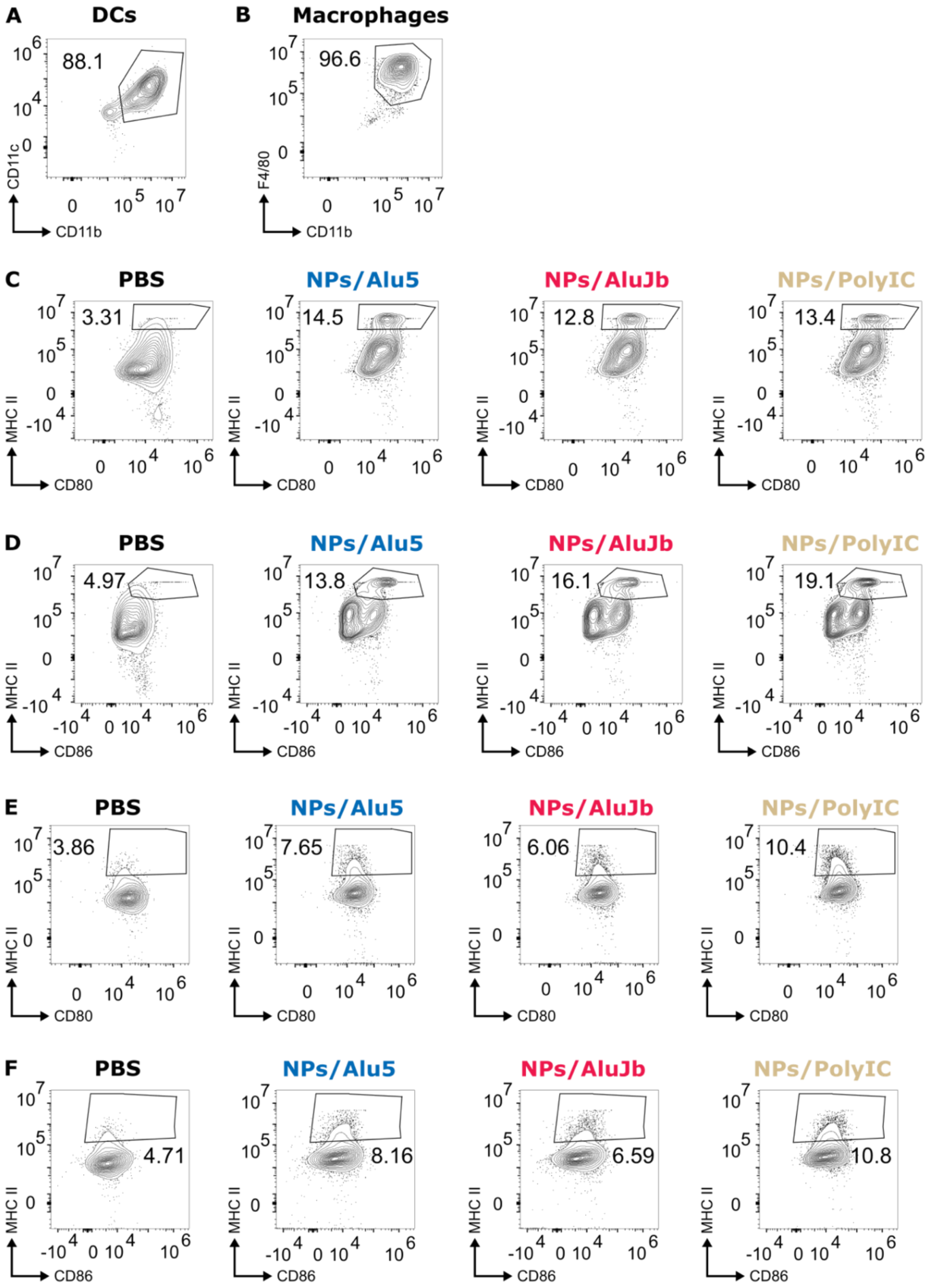

Figure S4. Representative flow cytometry plots from BMDC and BMDM Studies. A) DC purity. B) Macrophage purity. Representative flow cytometry plots of CD80 and MHC II levels in C) BMDMs and D) BMDCs. E) Representative flow cytometry plots of CD86 and MHC II levels in BMDCs. F) Representative flow cytometry plots of CD80 and MHC II levels in BMDMs. E) Representative flow cytometry plots of CD86 and MHC II levels in BMDMs. *n* = 3 per group and experiments were repeated twice.

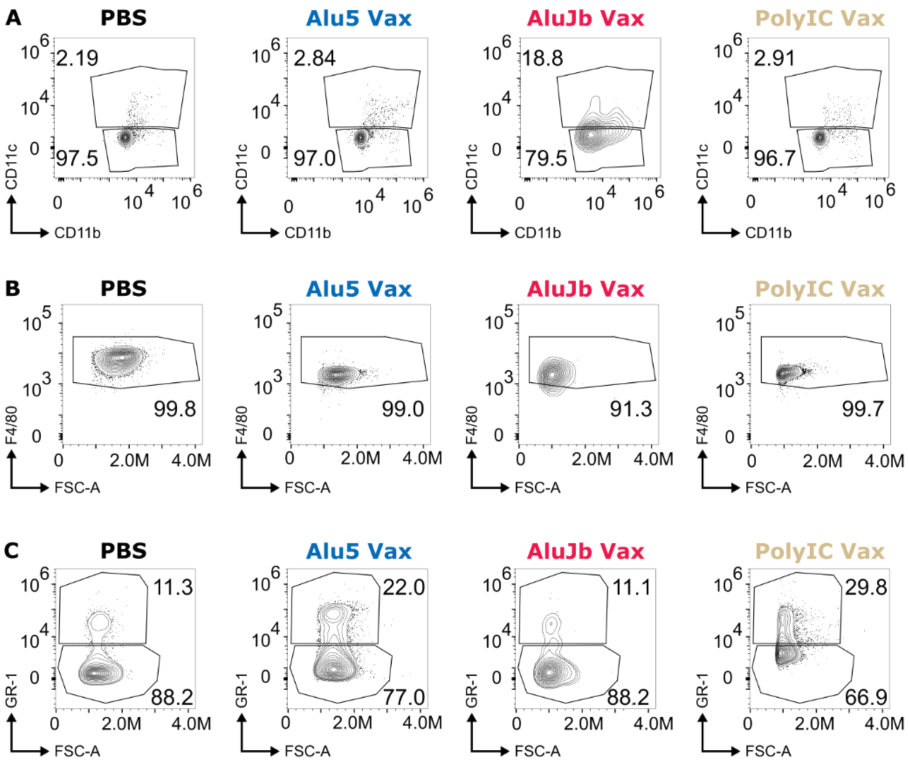

Figure S5. Representative flow cytometry plots of cell types in the draining lymph node. Identification of A) DCs, B) Macrophages, and C) Neutrophils. Data is represented as the frequency of the parent population. n = 3-5 per group.

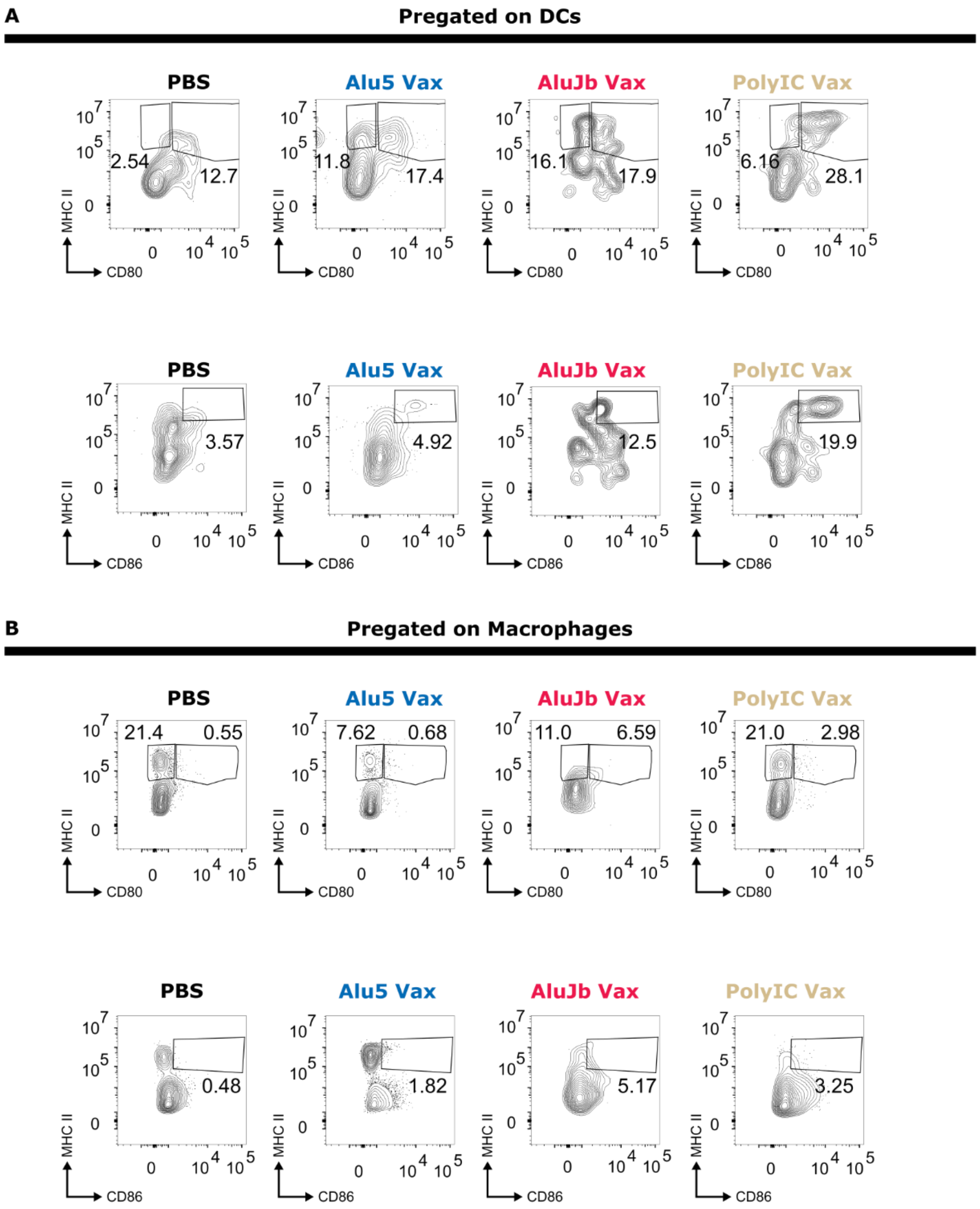

Figure S6. Representative flow cytometry of APC activation state in the draining lymph node. Plots showing A) DC and B) macrophage activation induced by indicated formulation. *n* = 3-5 per group

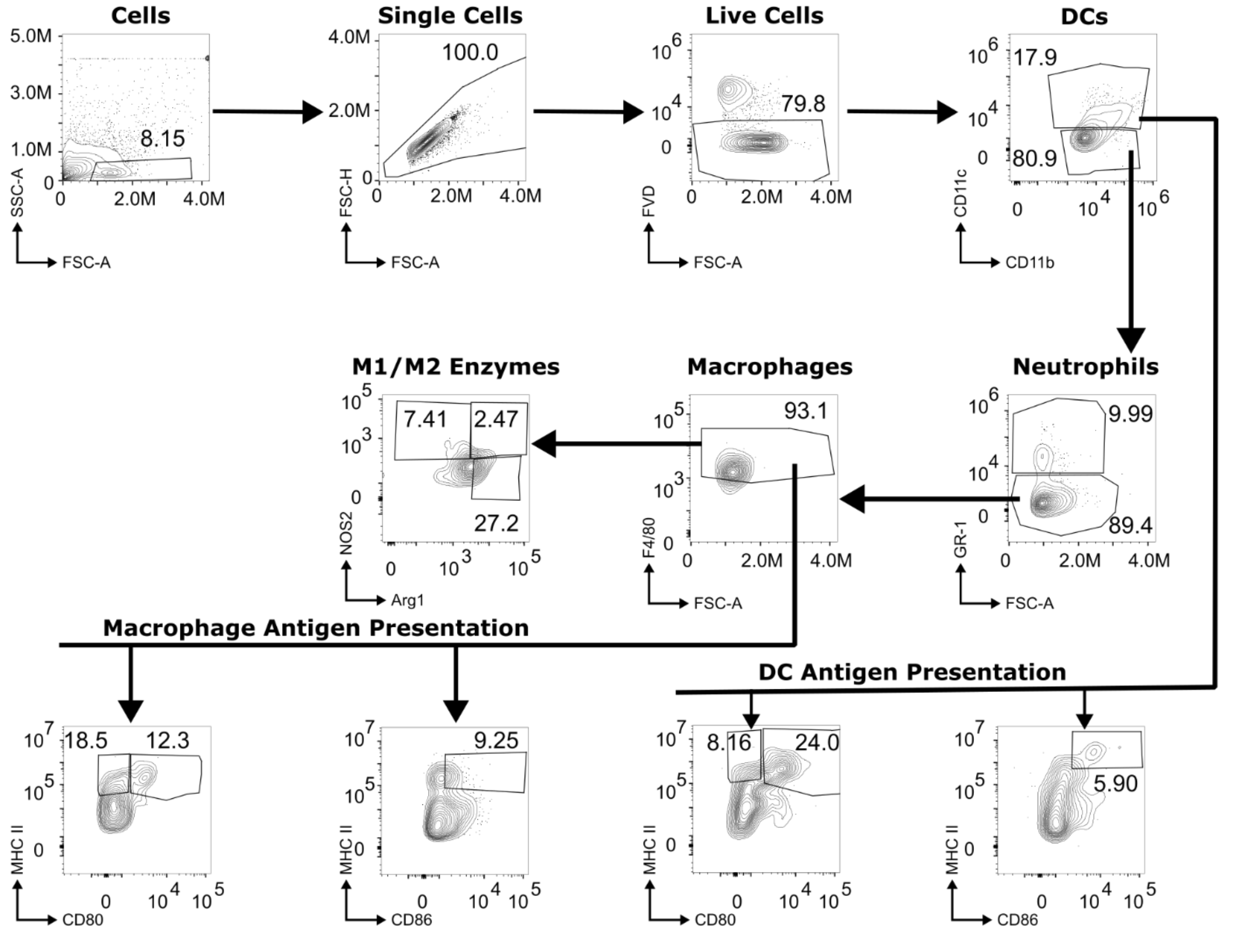

Figure S7. Draining lymph node flow cytometry gating. Representative flow cytometry plots showing how populations were gated in the draining lymph node.

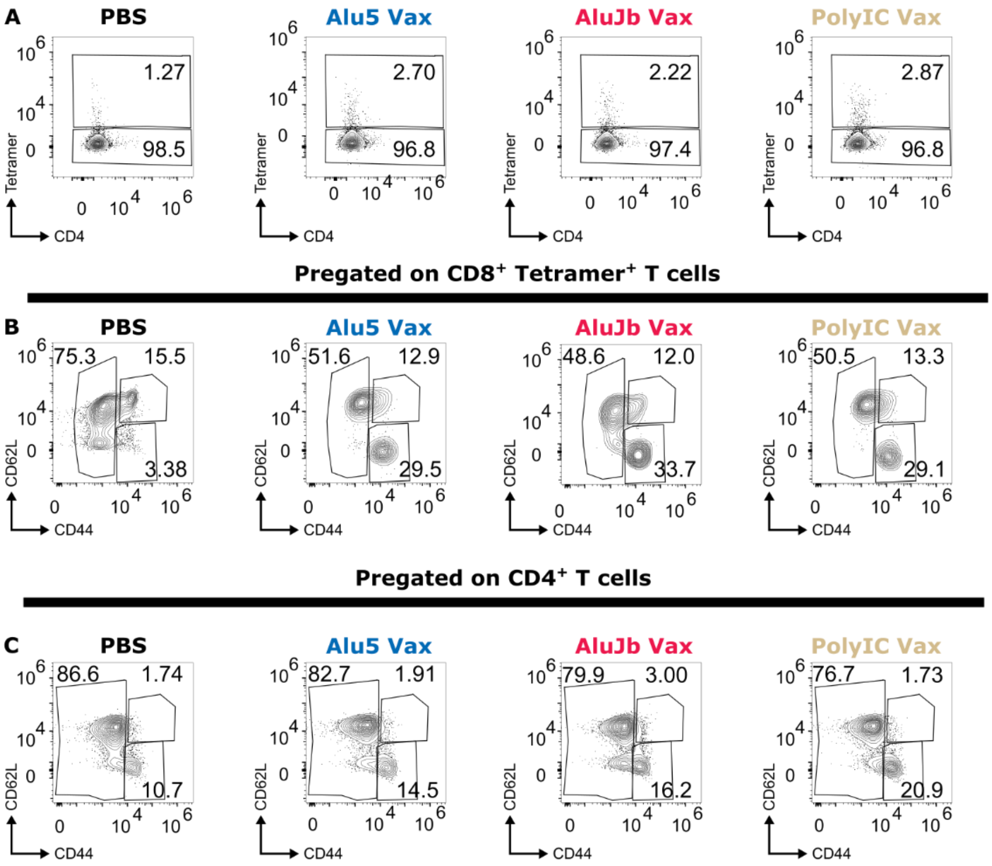

Figure S8. Representative flow cytometry plots of splenic populations. A) Percent tetramer-positive $CD8^+$ T cells for each treatment group. B) Representative flow cytometry plots of memory cell phenotype in $CD8^+$ T cells. T_{EM}: $CD44^+$ $CD62L^-$, T_{CM}: $CD44^+$ $CD62L^+$, Naive T cells $CD44^-$. C) Representative flow cytometry plots of memory cell phenotype in $CD4^+$ T cells. *n* = 7-10 per group.

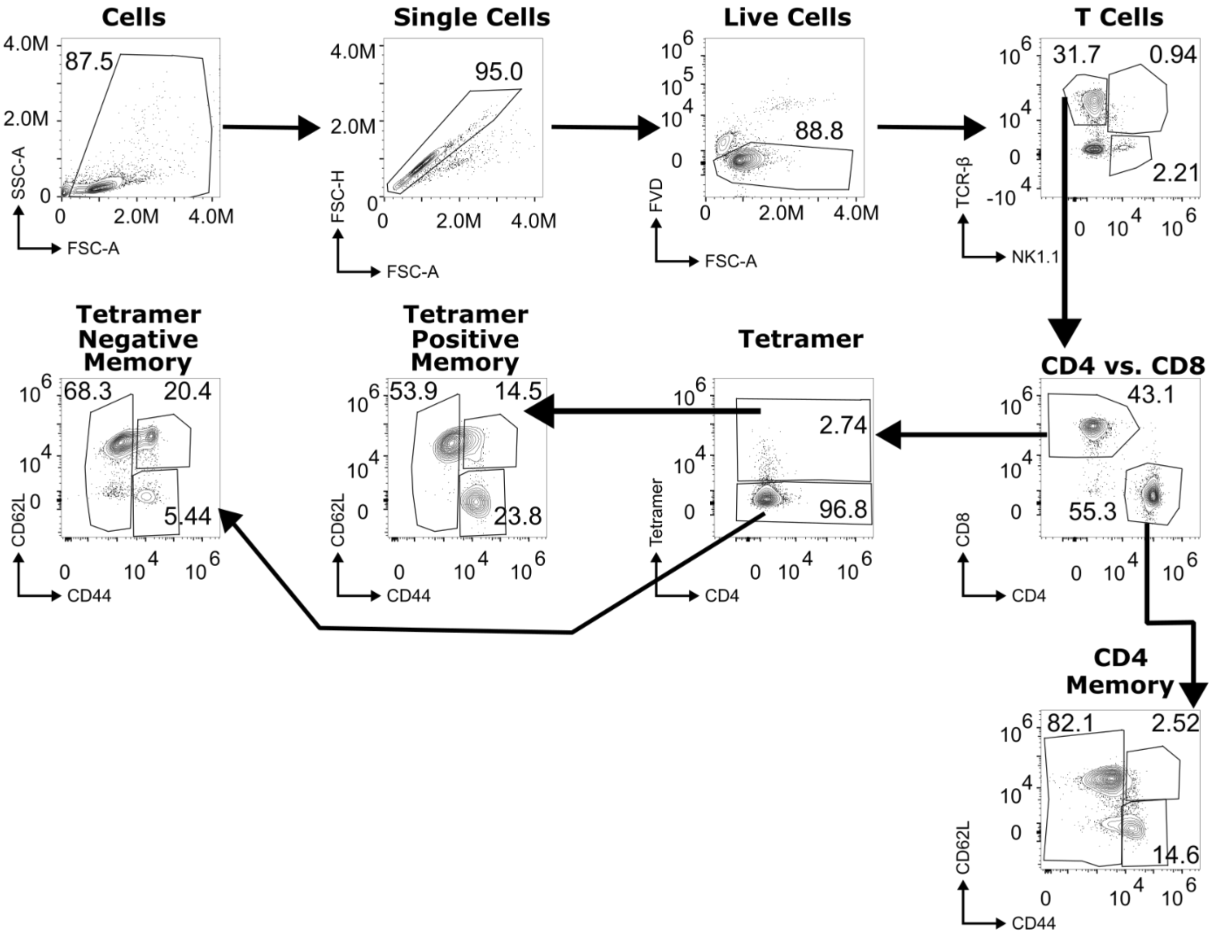

Figure S9. Gating for T cell analysis in spleen. Representative flow cytometry plots outlining how populations were gated in the spleen.
